# Empagliflozin reduces arrhythmogenic effects in rat neonatal and human iPSC-derived cardiomyocytes and improves cytosolic calcium handling at least partially independent of NHE1

**DOI:** 10.1038/s41598-023-35944-5

**Published:** 2023-05-29

**Authors:** Danúbia Silva dos Santos, Lauro Thiago Turaça, Keyla Cristiny da Silva Coutinho, Raiana Andrade Quintanilha Barbosa, Juliano Zequini Polidoro, Tais Hanae Kasai-Brunswick, Antonio Carlos Campos de Carvalho, Adriana Castello Costa Girardi

**Affiliations:** 1 Laboratório de Genética e Cardiologia Molecular, Faculdade de Medicina, Instituto do Coração (InCor), Hospital das Clínicas HCFMUSP, Universidade de São Paulo, Avenida Dr. Enéas de Carvalho Aguiar, 44 - Bloco II 10° Andar, São Paulo, 05403-900 Brazil; 2 Instituto de Biofísica Carlos Chagas Filho, Universidade Federal do Rio de Janeiro, Rio de Janeiro, RJ Brazil; 3 Centro de Tecnologia Celular, Instituto Nacional de Cardiologia, Rio de Janeiro, Brazil; 4 Centro Nacional de Biologia Estrutural e Bioimagem (CENABIO), Universidade Federal do Rio de Janeiro, Rio de Janeiro, RJ Brazil

**Keywords:** Cell biology, Physiology, Cardiology, Molecular medicine

## Abstract

The antidiabetic agent class of sodium-glucose cotransporter 2 (SGLT2) inhibitors confer unprecedented cardiovascular benefits beyond glycemic control, including reducing the risk of fatal ventricular arrhythmias. However, the impact of SGLT2 inhibitors on the electrophysiological properties of cardiomyocytes exposed to stimuli other than hyperglycemia remains elusive. This investigation tested the hypothesis that the SGLT2 inhibitor empagliflozin (EMPA) affects cardiomyocyte electrical activity under hypoxic conditions. Rat neonatal and human induced pluripotent stem cell (iPSC)-derived cardiomyocytes incubated or not with the hypoxia-mimetic agent CoCl_2_ were treated with EMPA (1 μM) or vehicle for 24 h. Action potential records obtained using intracellular microelectrodes demonstrated that EMPA reduced the action potential duration at 30%, 50%, and 90% repolarization and arrhythmogenic events in rat and human cardiomyocytes under normoxia and hypoxia. Analysis of Ca^2+^ transients using Fura-2-AM and contractility kinetics showed that EMPA increased Ca^2+^ transient amplitude and decreased the half-time to recover Ca^2+^ transients and relaxation time in rat neonatal cardiomyocytes. We also observed that the combination of EMPA with the Na^+^/H^+^ exchanger isoform 1 (NHE1) inhibitor cariporide (10 µM) exerted a more pronounced effect on Ca^2+^ transients and contractility than either EMPA or cariporide alone. Besides, EMPA, but not cariporide, increased phospholamban phosphorylation at serine 16. Collectively, our data reveal that EMPA reduces arrhythmogenic events, decreases the action potential duration in rat neonatal and human cardiomyocytes under normoxic or hypoxic conditions, and improves cytosolic calcium handling at least partially independent of NHE1. Moreover, we provided further evidence that SGLT2 inhibitor-mediated cardioprotection may be partly attributed to its cardiomyocyte electrophysiological effects.

## Introduction

Sodium-glucose cotransporter 2 (SGLT2) inhibitors, also known as gliflozins, are a novel class of antidiabetic agents that limit the reabsorption of glucose in the renal proximal tubule, leading to glycosuria and decreased hyperglycemia^1,2^. Cardiovascular outcome trials indicated that gliflozins induce a striking reduction in death risk from cardiovascular causes and heart failure hospitalization in patients with heart failure regardless of the presence^3–5^ or absence^6–8^ of type 2 diabetes. Numerous mechanisms have been proposed to explain the cardiovascular benefits of SGLT2 inhibitors supporting the concept that these agents act in a multifactorial multiorgan-beneficial fashion^9–13^. Notably, in the trials, the cardioprotection conferred by the gliflozins occurred early, and these benefits continued throughout the studies. Moreover, clinical trial data and meta-analysis revealed that SGLT2 inhibitors might reduce the risk of ventricular arrhythmias and sudden cardiac death in heart failure patients^12,14–16^. However, to the best of our knowledge, the effects of SGLT2 inhibitors on the cardiomyocyte electrophysiological properties, especially in response to stimuli other than hyperglycemia, have not been fully elucidated.

Ventricular arrhythmias can occur after acute ischemia due to the prolongation of action potential duration (APD)^17,18^, which may be associated with increased intracellular concentrations of sodium ([Na^+^]_i_) and calcium ([Ca^2+^]_i_) in the myocardium^19^. Elevated levels of [Ca^2+^]_i_ induce the spontaneous release of calcium from the sarcoplasmic reticulum (SR), leading to the generation of a transient inward current responsible for delayed afterdepolarizations (DADs)^20^. In this regard, Janse and colleagues have reported that increased spontaneous calcium release from SR observed in isolated myocytes is associated with ventricular tachycardia in rabbits with heart failure^21^. Furthermore, a reduction in [Ca^2+^]_i_ secondary to a decrease in [Na^+^]_i_ is related to the decline in spontaneous calcium release from SR and arrhythmic events^22–24^.

Molecular docking studies revealed that SGLT2 inhibitors have high affinity to the extracellular Na^+^ binding site of the Na^+^/H^+^ exchanger isoform 1 (NHE1)^25^, demonstrating that the direct cardiac effects of the gliflozins may be mediated via SGLT2-independent pathways. Cardiac NHE1 activity is relatively quiescent under basal conditions^26^, but it is stimulated by the intracellular acidification that occurs during myocardial ischemia resulting in a reversal of the Na^+^/Ca^2+^ exchanger and accumulation of calcium in the cytosol^27,28^. Thus, SGLT2 inhibitor-mediated NHE1 inhibition would act to lessen [Ca^2+^]_i_, thereby improving cytosolic calcium handling. However, it remains to be determined whether the effects of SGLT2 inhibitors on cytosolic calcium handling can be attributed to NHE1.

Based on these observations, the present study tested the hypothesis that empagliflozin (EMPA) affects cardiac electrical activity in non-hyperglycemic cardiomyocytes. More specifically, we investigated the direct impact of EMPA treatment on action potential recordings and spontaneous arrhythmogenic events in rat and human cardiomyocytes subjected to hypoxia. Furthermore, we investigated the effect of EMPA in association with the NHE1 inhibitor cariporide on cardiomyocytes Ca^2+^ transient, contraction-relaxation waves, and on posttranslational modulation of phospholamban, a key regulator of SR Ca^+^ sequestration^29^.

## Results

### Chemical-induced hypoxia prolongs action potential duration in cardiomyocytes

**Figure 1:**
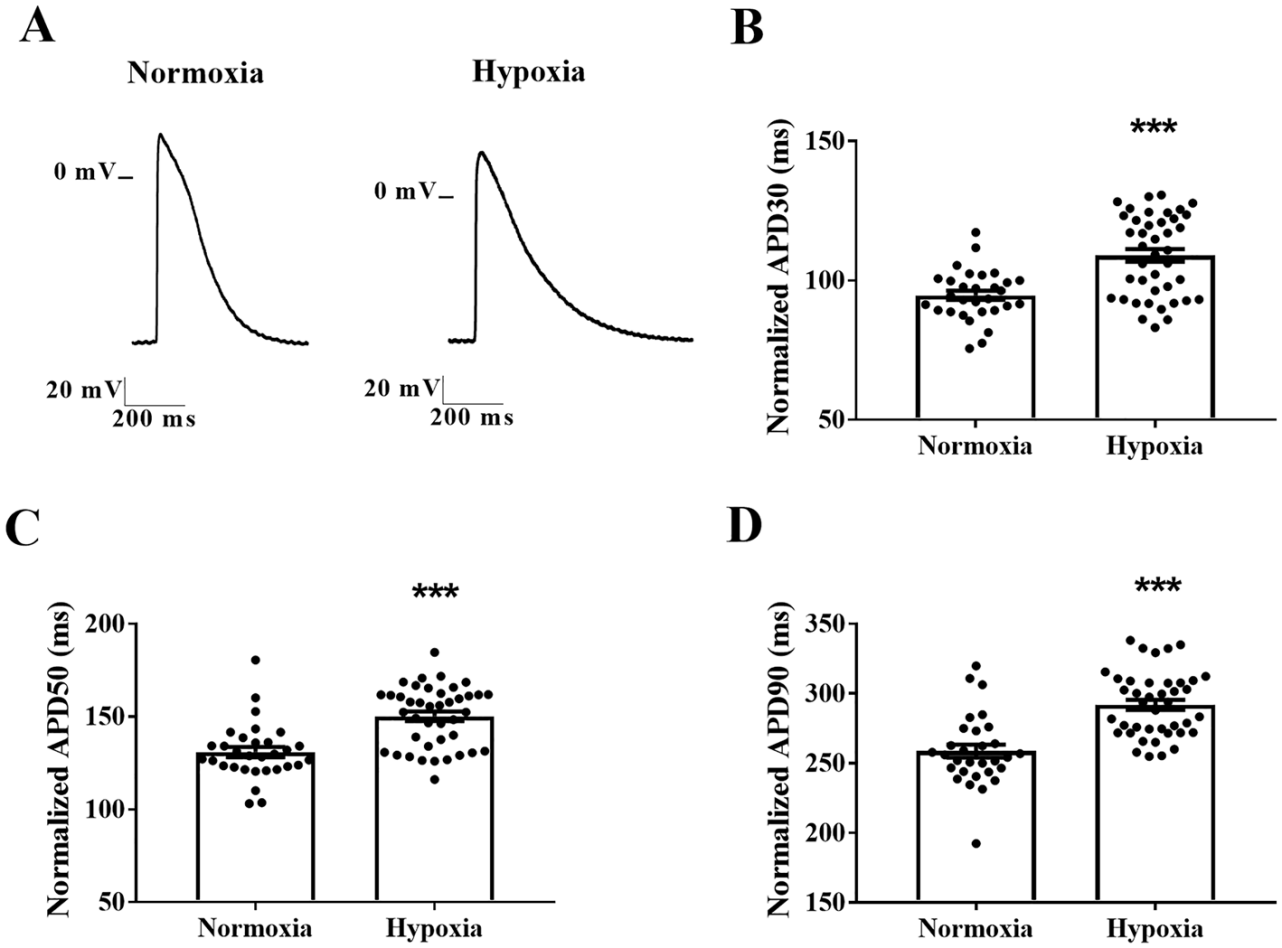
Chemical-induced hypoxia prolongs action potential duration in rat neonatal cardiomyocytes. (**A**) Representative action potential traces of cardiomyocytes subjected to normoxia or chemical hypoxia and treated with a vehicle. APD at 30% (**B**), 50% (**C**), and 90% (**D**) of repolarization corrected by the beat rate (*n* = 31–41). The points represent individual measurements, and the bars represent the mean ± SEM. The significance of differences was determined using the unpaired *t*-test or Mann–Whitney test. ***P < 0.001 versus normoxia.

**Figure 2:**
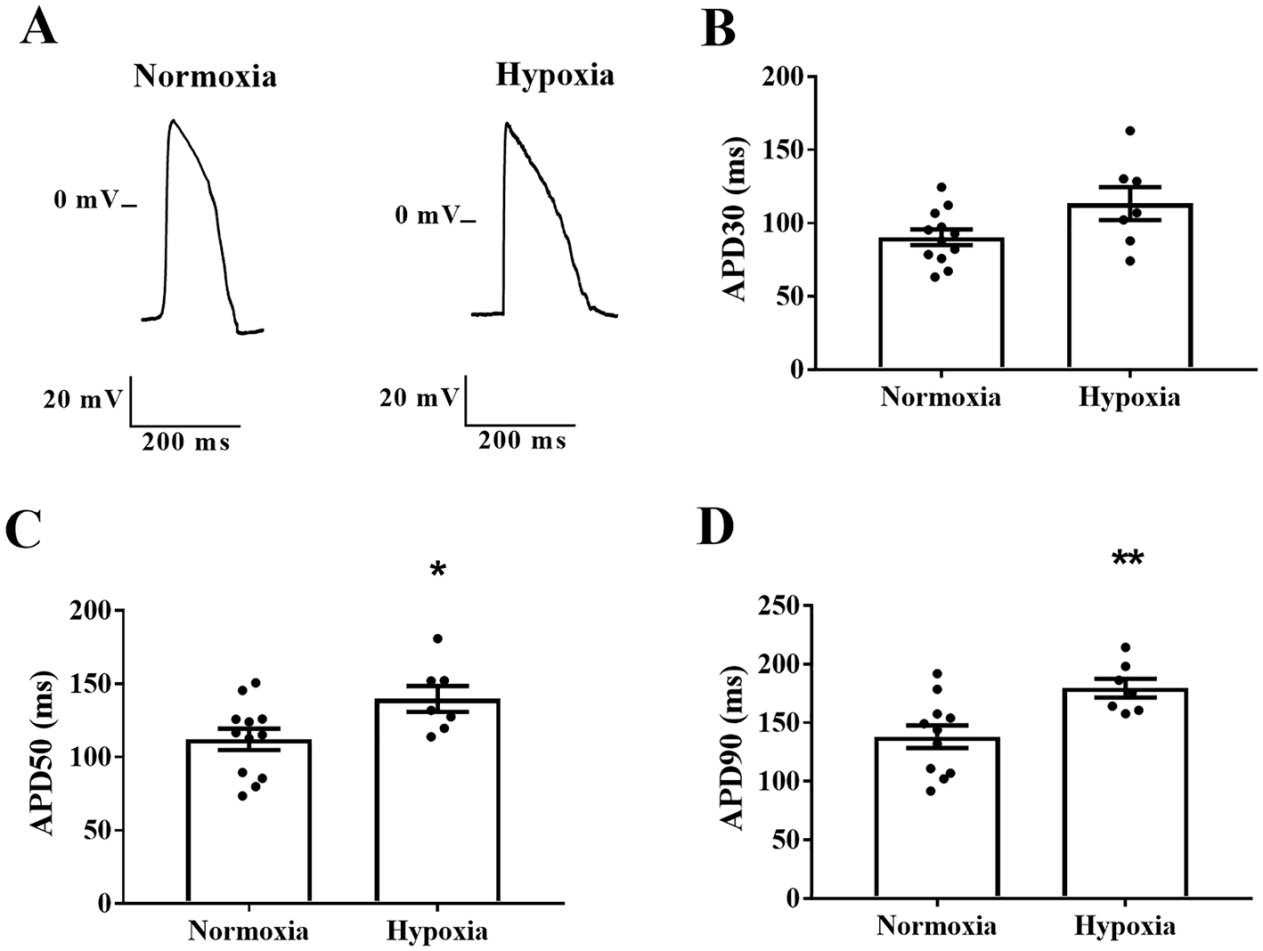
Chemical-induced hypoxia prolongs action potential duration in cardiomyocytes derived from human iPSCs. (**A**) Representative action potential traces of cardiomyocytes subjected to normoxia or chemical hypoxia and treated with a vehicle. Action potentials were evoked by pulses at 1 Hz. APD at 30% (**B**), 50% (**C**), and 90% (**D**) of repolarization (*n* = 7–12). The points represent individual measurements, and the bars represent the mean ± SEM. The significance of differences was determined using the unpaired *t*-test. *P < 0.05 and **P < 0.01 versus normoxia.

We assessed whether chemical hypoxia induces APD prolongation using an intracellular microelectrodes technique. Representative traces of the action potentials of rat cardiomyocytes subjected to normoxia or hypoxia are presented in Fig. 1A. We found that cardiomyocytes subjected to hypoxia had higher APDs at 30, 50, and 90% repolarization than in the vehicle group under normoxia (APD30: 109 ± 2 vs. 94 ± 2 ms, *P* < 0.001; APD50: 150 ± 3 vs. 131 ± 3 ms, *P* < 0.001; APD90: 292 ± 4 vs. 259 ± 5 ms, *P* < 0.001) (Fig. 1B–D). In addition, APD prolongation was observed in cardiomyocytes derived from human iPSCs subjected to chemical hypoxia when compared to normoxia (APD50: 140 ± 9 vs. 112 ± 7 ms, *P* < 0.05; APD90: 180 ± 8 vs. 138 ± 10 ms, *P* < 0.01) (Fig. 2).

### Empagliflozin reduced the action potential duration in rat neonatal cardiomyocytes

**Figure 3:**
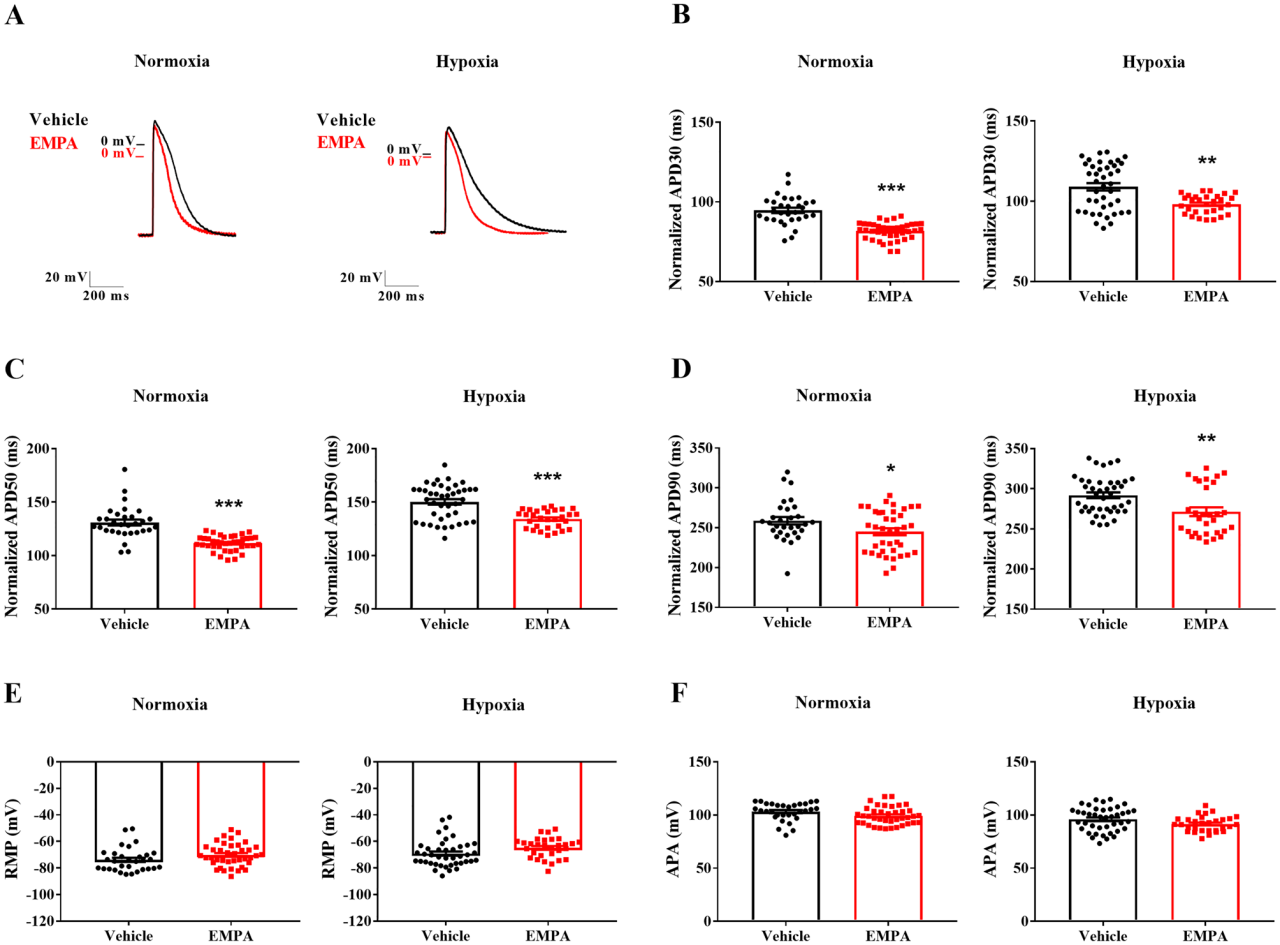
Empagliflozin reduced the action potential duration in rat neonatal cardiomyocytes under normoxic and hypoxic conditions. Action potential recordings in cardiomyocytes subjected to normoxia or chemical hypoxia and treated with vehicle or EMPA were obtained using the intracellular microelectrode technique. (**A**) Representative single-cell action potential traces. APD at 30% (**B**), 50% (**C**), and 90% (**D**) of repolarization corrected by the beat rate. **(E)** RMP. **(F)** APA. The points represent individual measurements, and the bars are the mean ± SEM (*n* = 30–41). The significance of differences was determined using the unpaired *t*-test or Mann–Whitney test. *P < 0.05, **P < 0.01, and ***P < 0.001 versus vehicle.

Action potentials were recorded after treatment with EMPA to test the hypothesis that the cardioprotective effects of SGLT2 inhibitors are associated with changes in cardiac electrical activity. Figure 3A compares representative action potentials recorded from rat neonatal ventricular myocytes subjected to normoxia or hypoxia and treated with vehicle or EMPA. Compared with vehicle-treated, EMPA-treated cardiomyocytes had shorter APDs at 30, 50, and 90% repolarization under normoxia (APD30: 81 ± 1 vs. 94 ± 2 ms, *P* < 0.001; APD50: 111 ± 1 vs. 129 ± 2 ms, *P* < 0.001; APD90: 244 ± 4 vs. 259 ± 4 ms, *P* < 0.05) and hypoxia (APD30: 98 ± 1 vs. 109 ± 2 ms, *P* < 0.01; APD50: 134 ± 1 vs. 150 ± 3 ms, *P* < 0.001; APD90: 271 ± 5 vs. 292 ± 4 ms, *P* < 0.01) (Fig. 3B–D). Subsequently, we evaluated the resting membrane potential (RMP) and amplitude (APA). As shown in Fig. 3E–F, both RMP and APA remained unchanged in vehicle- and EMPA-treated cardiomyocytes under either normoxic or hypoxic conditions.

### Empagliflozin reduced arrhythmogenic events in rat neonatal cardiomyocytes

**Figure 4:**
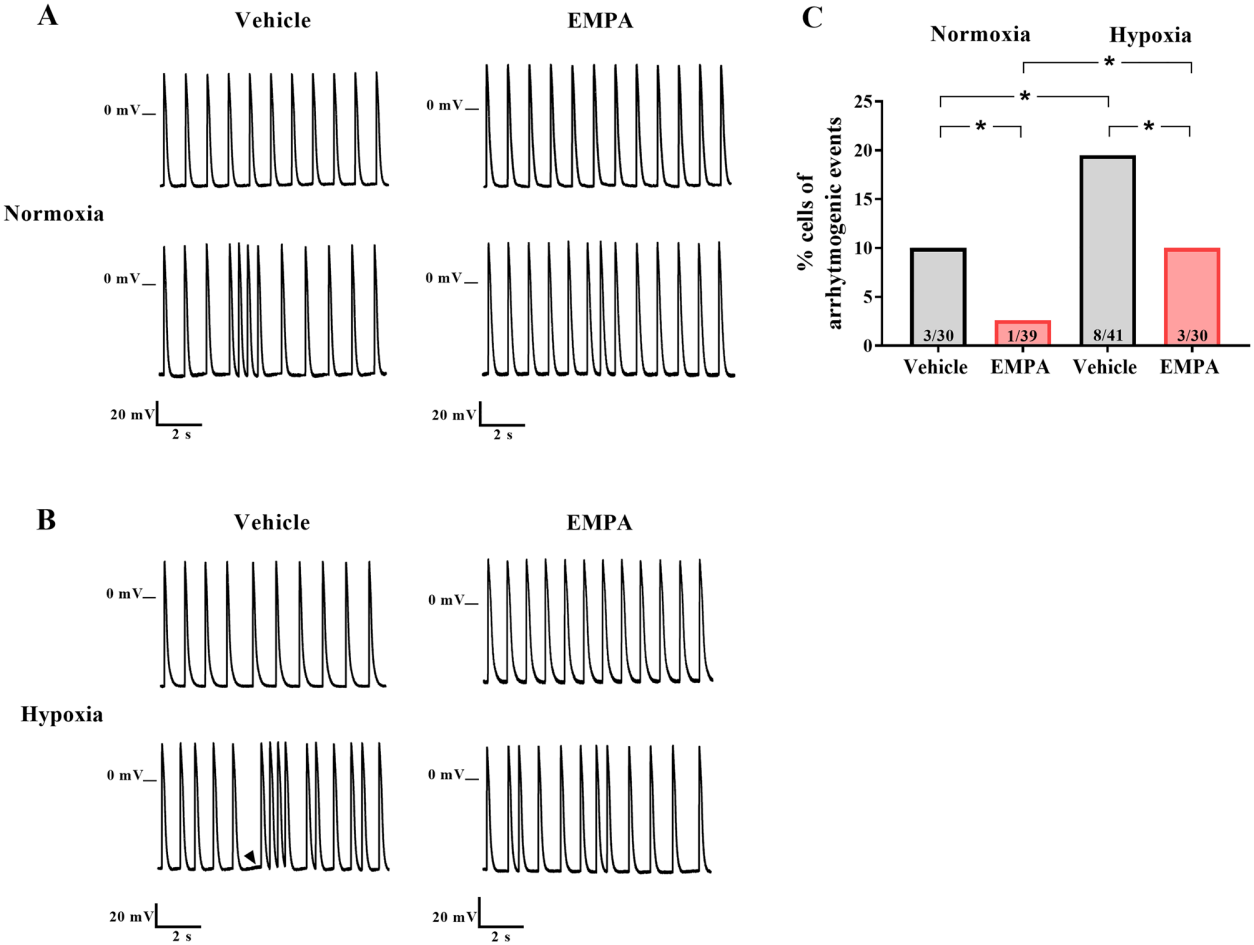
Empagliflozin reduced arrhythmogenic events in rat neonatal cardiomyocytes. Action potentials were recorded from spontaneously beating rat neonatal cardiomyocytes using the intracellular microelectrode technique. The occurrence of arrhythmogenic events (irregular and/or -triggered-like activities) of beating cells was assessed. Representative recordings show a normal beating rhythm (top) and irregular beating pattern (bottom) of cardiomyocytes treated with vehicle (right) or EMPA (left) under normoxia (**A**) and hypoxia (**B**) conditions. The black arrow indicates a triggered-like activities. (**C**) The proportion of cells showing arrhythmogenic events. Number of cells with arrhythmogenic events (total cells): normoxia, vehicle *n* = 3^30^ and EMPA *n* = 1^39^; hypoxia, vehicle *n* = 8^41^ and EMPA *n* = 3^30^. *P* values were calculated using Fisher's exact test. *P < 0.05.

Figure 4 illustrates cells that presented arrhythmogenic events while acquiring spontaneous action potential recordings. Figure 4A,B depicts normal and irregular beating rhythm recordings of action potentials in cardiomyocytes treated with vehicle or EMPA under normoxia and hypoxia conditions. Cardiomyocytes treated with vehicle and EMPA under hypoxia exhibited a higher proportion of cells with arrhythmogenic events when compared to normoxia (Fig. 4C). Additionally, the arrhythmogenic events occurred in a lower proportion of cardiomyocytes treated with EMPA than the vehicle group under normoxia (3% vs. 10%) and hypoxia (10% vs. 19%) (Fig. 4C). Moreover, we observed the presence of triggered-like activities in two of the eight cells under hypoxia treated with vehicle (Fig. 4B). Overall, our data provide strong evidence that EMPA reduces the occurrence of arrhythmogenic events in hypoxic cardiomyocytes, including triggered-like activities..

### Empagliflozin reduced the action potential duration and arrhythmogenic events in cardiomyocytes derived from human iPSCs

**Figure 5:**
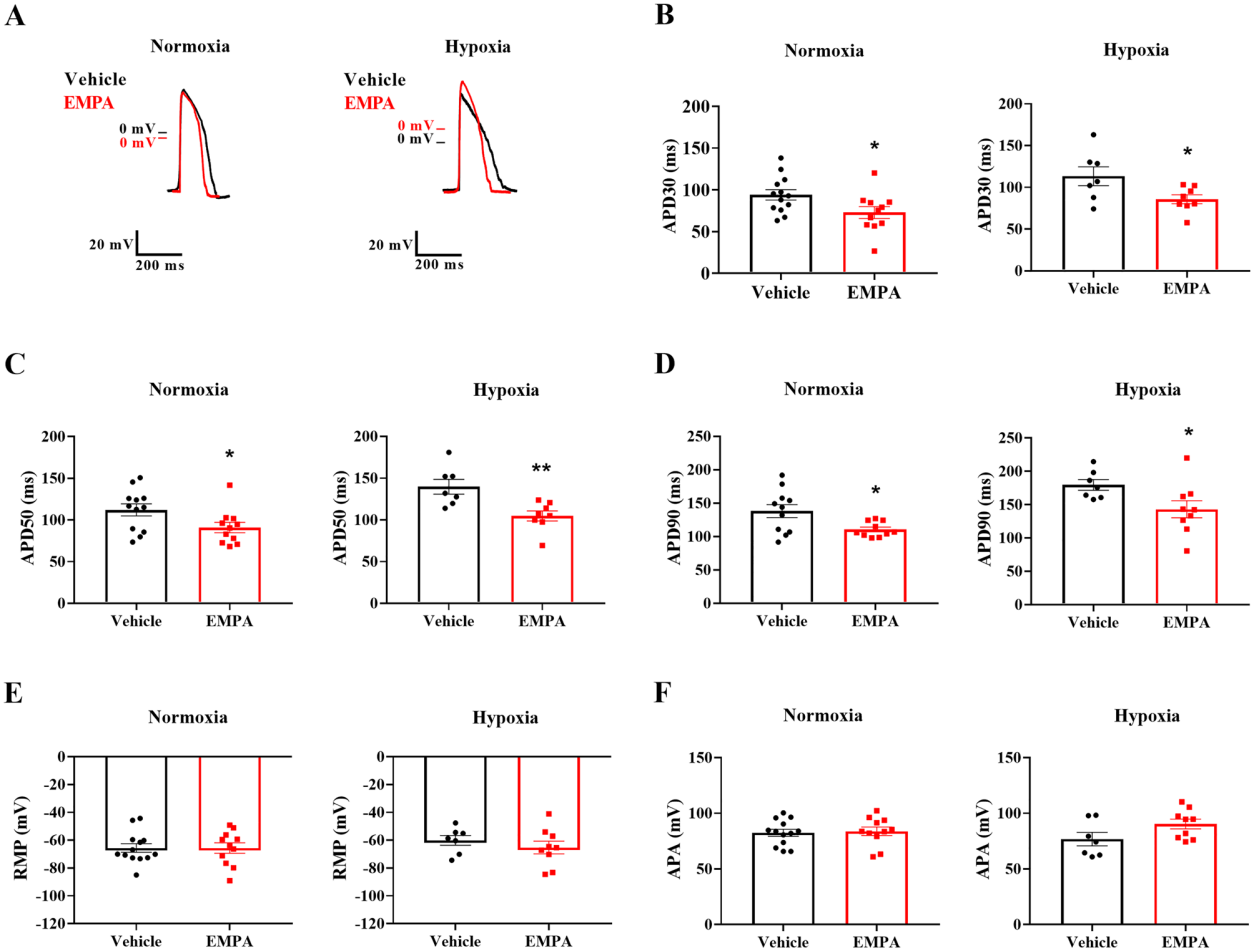
Empagliflozin decreased the action potential duration in cardiomyocytes derived from human iPSCs under normoxic and hypoxic conditions. Action potential recordings in cardiomyocytes derived from humaniPSCs subjected to normoxia or chemical hypoxia and treated with vehicle or EMPA were obtained using the intracellular microelectrode technique. Action potentials were evoked by pulses at 1 Hz. (**A**) Representative single-cell action potential traces. Action potential duration (APD) at 30% **(B)**, 50% **(C)**, and 90% **(D)** of repolarization. (**E**) RMP. (**F**) APA. The points represent individual measurements, and the bars are the mean ± SEM (*n* = 7–13). The significance of differences was determined using the unpaired *t*-test or Mann–Whitney test. *P < 0.05 and **P < 0.01 versus vehicle.

To exclude the hypothesis that the effects of EMPA on cardiac electrical activity are strictly limited to rat neonatal cardiomyocytes, action potentials were acquired using cardiomyocytes derived from human iPSCs. Representative traces of action potentials of cardiomyocytes derived from iPSCs under normoxia or hypoxia and treated with vehicle or EMPA are presented in Fig. 5A. EMPA-treated cardiomyocytes had shorter APDs at 30, 50, and 90% repolarization than the vehicle group under normoxia (APD30: 73 ± 7 vs. 94 ± 6 ms, *P* < 0.05; APD50: 91 ± 6 vs. 112 ± 7 ms, *P* < 0.05; APD90: 111 ± 4 vs. 138 ± 10 ms, *P* < 0.05) and hypoxia (APD30: 86 ± 5 vs. 113 ± 11 ms, *P* < 0.05; APD50: 105 ± 6 vs. 140 ± 9 ms, *P* < 0.01; APD90: 143 ± 13 vs. 180 ± 8 ms, *P* < 0.05) (Fig. 5B–D). In addition, no differences in RMP and APA between vehicle and EMPA-treated cardiomyocytes were observed under either experimental condition (Fig. 5E–F).

**Figure 6:**
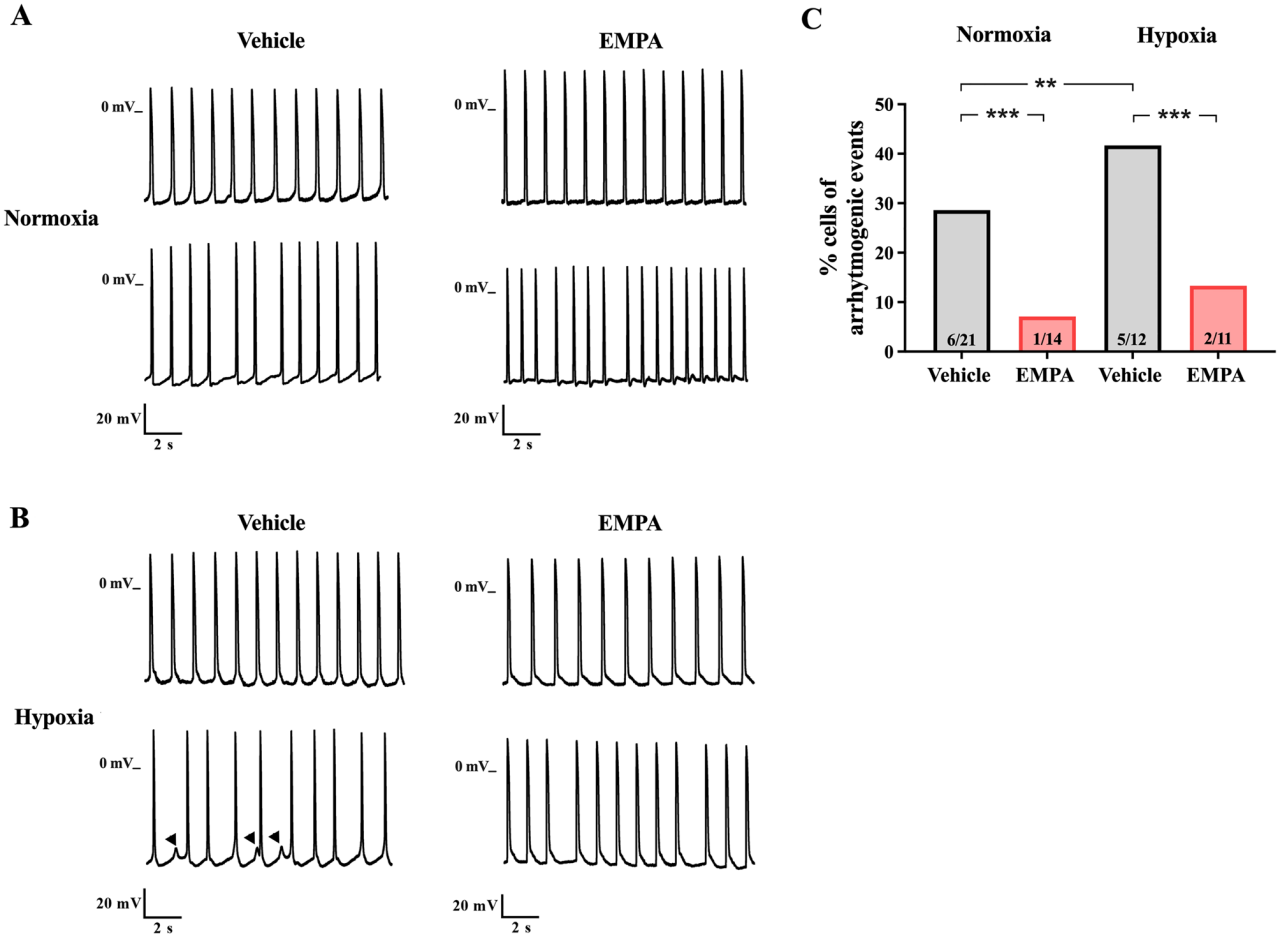
Empagliflozin reduced arrhythmogenic events in cardiomyocytes derived from human iPSCs. Action potentials were recorded from spontaneously beating cardiomyocytes derived from human iPSCs using the intracellular microelectrodes technique. The occurrence of arrhythmogenic events (irregular and/or DAD-like events) of beating cells was assessed. Representative recordings show a normal beating rhythm (top) and irregular beating pattern (bottom) of cells treated with vehicle (right) or EMPA (left) under normoxia (**A**) and hypoxia (**B**) conditions. The black arrows indicate DAD-like events. (**C**) The proportion of cells showing arrhythmogenic events. Number of cells with arrhythmogenic events (total cells): normoxia, vehicle *n* = 6^21^ and EMPA *n* = 1^14^; hypoxia, vehicle *n* = 5^12^ and EMPA *n* = 2^15^. *P* values were calculated using Fisher's exact test. **P < 0.01 and ***P < 0.001.

Figure 6A–B shows the presence of normal and irregular beating rhythm recordings of action potentials in iPSCs-derived cardiomyocytes under normoxia or hypoxia and treated with vehicle or EMPA. Vehicle-treated cardiomyocytes under hypoxia exhibited a higher proportion of cells with arrhythmogenic events compared to the vehicle group under normoxia (Fig. 6C). However, no differences in arrhythmogenic events were observed between cardiomyocytes treated with EMPA under normoxia and hypoxia (Fig. 6C). Notably, the arrhythmogenic events were also less frequent in EMPA-treated cells than in the vehicle group under both conditions (normoxia, 7% vs. 29%; hypoxia, 18% vs. 42%) (Fig. 6C). Furthermore, we observed DAD-like events, which occur after complete repolarization (phase 3) of the action potential^30^, in one of five vehicle-treated cardiomyocytes subjected to hypoxia (Fig. 6B).

### Empagliflozin influences Ca^2+^ transient and contractility in rat neonatal cardiomyocytes at least partially independent of NHE1

**Figure 7:**
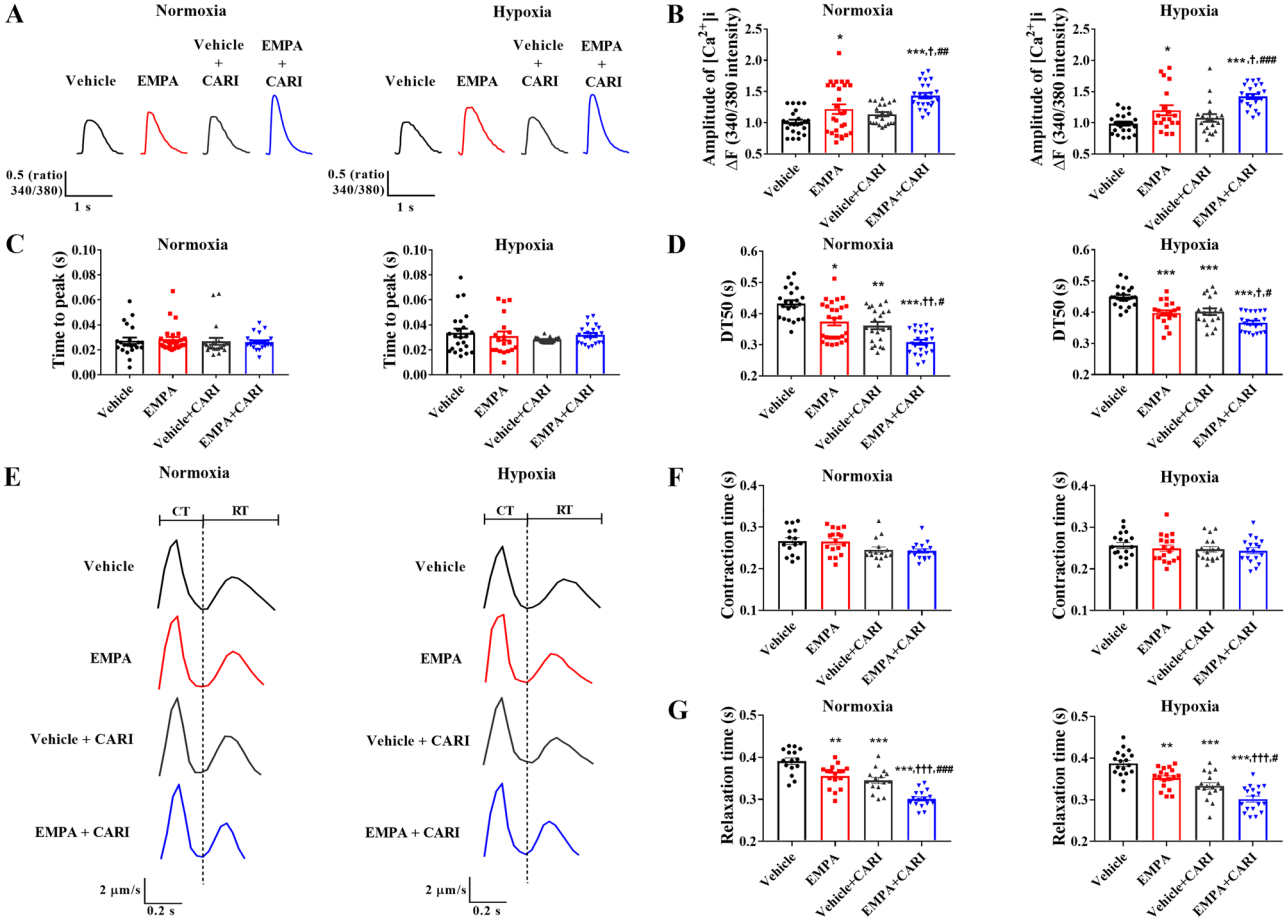
Empagliflozin influences the calcium transient and contractility in rat neonatal cardiomyocytes partially independent of NHE1. [Ca^2+^]_i_ transient traces and contractility recordings were performed in cardiomyocytes subjected to normoxia or chemical hypoxia and treated with vehicle or EMPA, in combination or not with CARI. (**A**) Representative [Ca^2+^]_i_ transient traces. (**B**) [Ca^2+^]_i_ transient amplitude. (**C**) Time to the peak. (**D**) Decreasing time to 50% relaxation (DT50). **(E)** Representative contraction-relaxation waves. (**F**) Contraction time. (**G**) Relaxation time. The points represent individual measurements, and the bars represent the mean ± SEM (*n* = 15–27). Differences were determined using one-way ANOVA followed by post hoc Tukey analysis or Kruskal–Wallis test, both with multiple comparisons with 95% confidence. *P < 0.05, **P < 0.01, and ***P < 0.001 versus vehicle; ^†^*P* < 0.05, ^††^*P* < 0.01, and ^†††^*P* < 0.001 versus EMPA; ^#^*P* < 0.05 and ^###^*P* < 0.001 versus vehicle + CARI. CT: contraction time; RT: relaxation time.

Ca^2+^ transients and contraction-relaxation cycle waves were recorded in rat cardiomyocytes treated with vehicle or EMPA alone or combined with the NHE1 inhibitor cariporide (CARI). Figure 7A shows representative traces of [Ca^2+^]_i_ transients from cells subjected to normoxia and hypoxia. Baseline Ca^2+^ levels were similar in all groups in normoxic (vehicle: 0.720 ± 0.006 µM; EMPA: 0.683 ± 0.015 µM; vehicle + CARI: 0.697 ± 0.008 µM; EMPA + CARI: 0.716 ± 0.010 µM) and hypoxic (vehicle: 0.682 ± 0.008 µM; EMPA: 0.675 ± 0.006 µM; vehicle + CARI: 0.694 ± 0.010 µM; EMPA + CARI: 0.684 ± 0.012 µM) conditions. Compared with vehicle treatment, 24 h of EMPA exposure significantly increased the amplitude and reduced the half-time for the recovery of [Ca^2+^]_i_ transients in cardiomyocytes subjected to normoxia and hypoxia (Fig. 7B,D). No difference in the time to peak was observed among the experimental groups (Fig. 7C). Although no difference in Ca^2+^ amplitude was observed (Fig. 7B), the half-time for the recovery of [Ca^2+^]_i_ transients in the vehicle + CARI group was significantly shorter than in the vehicle group and similar to the EMPA group (Fig. 7D) under normoxia or hypoxia. Interestingly, the changes observed in Ca^2+^ amplitude and the half-time for the recovery of [Ca^2+^]_i_ transients were more pronounced in the EMPA + CARI group compared to the other three groups of cells under both conditions (Figs. 7B,D).

Subsequently, we analyzed the effects of EMPA in the presence or absence of CARI on the contractility of rat cardiomyocytes. Representative traces of the contraction-relaxation cycles of cardiomyocytes treated or untreated with EMPA and cariporide under normoxia and hypoxia are shown in Fig. 7E. Although no difference in contraction phase time was observed among the four experimental groups (Fig. 7F), EMPA-treated cardiomyocytes have shorter relaxation times than the vehicle group under normoxia and hypoxia (Fig. 7G). Likewise, vehicle + CARI-treated cells displayed lower relaxation times than the vehicle group. No differences in the relaxation times were found between vehicle + CARI and EMPA-treated cells (Fig. 7G). Moreover, the relaxation time after treatment with EMPA and cariporide was shorter than in the three groups (Fig. 7G).

**Figure 8:**
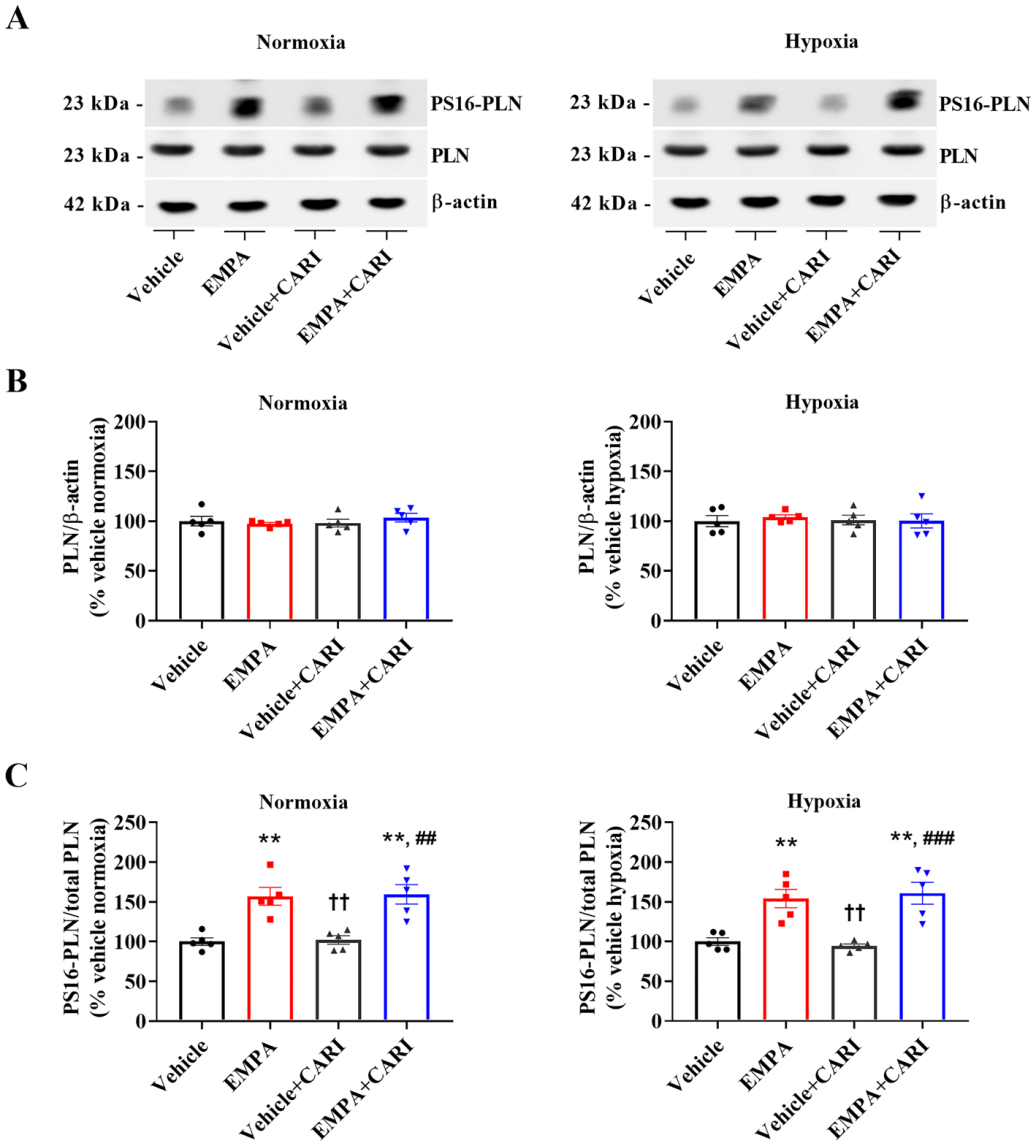
Empagliflozin increases phospholamban phosphorylation at serine 16 in rat neonatal cardiomyocytes. (**A**) Representative immunoblots following SDS-PAGE from rat neonatal cardiomyocytes subjected to normoxia or hypoxia, treated with vehicle or EMPA alone or in combination with the NHE1 inhibitor CARI and probed with antibodies against (PS16-PLN, total PLN, and actin, which was used as an internal control (unedited gels can be seen in Supplemental Material, Fig. S5). Graphical representation of the relative expression of (**B**) total PLN and (**C**) the ratio of PS16-PLN to total PLN. The points represent individual measurements, and the bars represent the mean ± SEM (*n* = 5). Differences were determined using one-way ANOVA followed by post hoc Tukey analysis with multiple comparisons with 95% confidence. **P < 0.01 versus vehicle; ^††^*P* < 0.01 versus EMPA; ^###^*P* < 0.001 versus vehicle + CARI.

To explore additional mechanisms underlying the effects of EMPA on cytosolic Ca^2+^ handling, we performed immunoblotting to assess the protein levels of phospholamban phosphorylated at serine 16 (PS16-PLN) and total phospholamban (PLN) as well as the ratio of PS16-PLN to total PLN. As seen in Fig. 8A–C, EMPA-treated cardiomyocytes displayed higher levels of the PS16-PLN to total PLN than in the vehicle and vehicle + CARI-treated cells under normoxia (EMPA: 157 ± 11% and EMPA + CARI: 159 ± 12% vs. vehicle: 100 ± 5% and vehicle + CARI: 102 ± 5%). Similar results were observed in hypoxia (EMPA: 154 ± 12% and EMPA + CARI: 161 ± 14% vs. vehicle: 100 ± 5%; vehicle + CARI: 94 ± 3%). However, no differences in these parameters were observed between vehicle and vehicle + CARI-treated cells (Figs. 8A–B). Moreover, no changes in total PLN expression were observed among the experimental groups (Fig. 8C).

## Discussion

This study provides novel experimental evidence that EMPA directly affects the electrophysiological properties of cardiomyocytes under non-hyperglycemic conditions. More specifically, we found that EMPA reduces arrhythmogenic events in rat and human cardiomyocytes under hypoxia, demonstrated by a decrease in the percentage of cells with irregular beating rhythms and/or absence of DADs or triggered activity in the electrical potential records. These anti-arrhythmogenic effects were accompanied by a decline in the action potentials' duration and a decrease in the half-time for the recovery of [Ca^2+^]_i_ transients. Moreover, EMPA increased cardiomyocyte relaxation kinetics under either normoxia or chemical hypoxia. Notably, the effects of the combination of EMPA with the NHE1 inhibitor cariporide on calcium transient and cardiomyocyte contractility were more pronounced than those of either EMPA or cariporide alone.

It has been postulated that SGLT2 inhibitors may reduce the incidence of fatal arrhythmias at least partially due to an effect on the cardiac APD^11,12^. Therefore, given the paucity of evidence supporting that SGLT2 inhibitors induce shortening of the cardiac APD in response to pathological stimuli other than hyperglycemia, we investigated whether EMPA affects APD in rat and human iPSCs-derived cardiomyocytes subjected to chemical hypoxia. We observed that the hypoxia-mimetic agent CoCl_2_ prolonged APD in rat and human cardiomyocytes compared to normoxia. Similar to what has been previously observed in cardiomyocytes isolated from diabetic rats^31^, we found that EMPA reverses the APD prolongation of hypoxic cardiomyocytes. Remarkably, EMPA also reduced the APD of rat cardiomyocytes under normoxia, albeit to a lesser extent than in hypoxia. In addition, despite differences in distribution and density of ion channels responsible for action potential morphology between rats and humans^32^, EMPA treatment also reduced the APD of cardiomyocytes derived from human iPSCs under both normoxia and hypoxia. The unexpected finding that EMPA affects the APD duration under normoxia may be attributed to the immature characteristics of neonatal and iPSCs-derived cardiomyocytes. Noteworthy, neonatal murine ventricular myocytes display longer APD than their adult counterpart^33^, at least partly by a lesser contribution of the fast hyperpolarizing current Ito. This transient outward K^+^ current is rapidly activated and deactivated after membrane depolarization^34^ and is also minimal in human iPSCs-derived cardiomyocytes^35^. Ito downregulation and APD prolongation are also observed in human failing hearts^36^ and rat-infarcted hearts^37^. Our assumption regarding the limitation of our cellular models, i.e., the immature characteristics of neonatal and iPSCs-derived compared to adult cardiomyocytes, is strengthened by the study of Xue et al.^38^, who showed that Langendorff’s perfusion with EMPA reduces APD, affects Ca^2+^ decay, attenuates arrhythmias, and decreases DAD events in infarcted hearts but does not affect APD and Ca^2+^ transients in healthy hearts from adult mice.

Furthermore, the action potential shortening induced by EMPA was already apparent at APD30, suggesting that the ion channel targeted by EMPA is recruited in the early phase of the action potential. As the increase in late Na^+^ current produced by the cardiac voltage-gated sodium channel (Na_v_1.5) is a persistent depolarizing force during the plateau that opposes repolarization currents and prolongs the action potential^39^, the shorter EMPA-induced APD30 observed by our study may be associated with the inhibition of late Na^+^ current. Indeed, EMPA has been reported to inhibit late Na^+^ currents in isolated cardiomyocytes^31,40–42^, at least partially due to the direct binding of SGLT2 inhibitors to Na_v_1.5^40^. Nevertheless, since EMPA also reduces APD50 and APD90, another potential target of this gliflozin might be the delayed K^+^ current, which is the primary outward current responsible for phase 3 of cardiac repolarization^43,44^. This hypothesis is strengthened by the recent report of Karpushev and colleagues in which EMPA upregulated rapid and slow components of the delayed rectifier K^+^ current in zebrafish ventricular cardiomyocytes^45^. However, as those hypotheses were not directly tested in this study, future studies are necessary to explore whether Na_v_1.5 and/or delayed rectifier K^+^ channel impact the observed SGLT2 inhibitor effect on rat and human cardiomyocyte action potential shortening.

Changes in the morphology of action potentials can lead to arrhythmias and, in more severe cases, sudden cardiac death^17,46,47^. Since the single-cell model has been used to assess cardiac rhythm disorders^41^, we evaluated the direct effects of SGLT2 inhibitors on arrhythmogenic events observed in spontaneous action potential recordings. We noticed that EMPA reduces the percentage of rat and human cardiomyocytes with irregular rhythm beating under normoxic and hypoxic conditions. Notably, DAD-like events were not seen in cardiomyocytes treated with EMPA, strongly suggesting that SGLT2 inhibitors may prevent arrhythmia by altering calcium handling, thus improving cardiomyocyte physiology. As arrhythmias evoked by DADs are associated with abnormal changes in [Ca^2+^]_i_^17,30,48,49^, we investigated whether EMPA affects Ca^2+^ transients. We observed that EMPA treatment increased the amplitude and reduced the half-time for the recovery of [Ca^2+^]_i_ transients and the relaxation time under hypoxic and normoxic conditions. In support of our results, Azam and colleagues showed that EMPA mitigated cardiac Ca^2+^ dysregulation in an ex vivo model of ischemia–reperfusion^50^. In addition, EMPA has also been reported to increase the Ca^2+^ transient amplitude in failing ventricular cardiomyocytes of the transverse aortic constriction (TAC) heart failure model and healthy cells^42^. Notably, we observed a significant increase of PLN phosphorylation at serine 16 residue, a common regulatory mechanism for upregulating SERCA activity^51^. The finding that SERCA2 overexpression in rabbit cardiomyocytes is associated with lower after-contraction events^52^ further supports that the observed improvement of Ca^2+^ decay kinetics and potential protection against SR Ca^2+^ leak may be a relevant mechanism accounting for decreased DAD-like events. It is worth mentioning that Ca^2+^ decay acceleration induced by EMPA might not only contribute to protecting cardiomyocytes from arrhythmogenic events, but it might also be a vital component accounting for the improvement of cardiac relaxation ability observed in experimental and clinical SGLT2 inhibitor studies^53–55^.

Intriguingly, we noted that EMPA-treated cardiomyocytes did not accelerate contraction phase kinetics, albeit presenting higher and faster Ca^2+^ transients. One potential explanation to reconcile these results is that EMPA may induce posttranslational modification of contractile proteins and consequently modulate the sensitivity of these proteins to calcium. This hypothesis arises from the Pabel and colleagues' study, which showed that EMPA acutely reduced the diastolic tension of isolated cardiac muscles from patients with preserved ejection fraction (HFpEF) without affecting Ca^2+^ transient parameters^53^. These authors observed that the phosphorylation pattern of contractile proteins was rescued by acute EMPA treatment in human and rat HFpEF samples. Interestingly, the increase in protein phosphorylation induced by EMPA was observed for some contractile proteins, even in non-failing human and murine hearts^53^. Consistent with Pabel's study, Kojin and colleagues found that acute EMPA treatment of heart tissue from obese rats, HFpEF rats, and HFpEF patients was associated with an improvement of passive cardiomyocyte stiffness and an increase of phosphorylation status of different contractile proteins at consensus sites for proteins kinase A (PKA) and G (PKG), including the cardiac troponin I phosphorylation at serine 23/24 residues^56^. As demonstrated by Rao and colleagues, phosphorylation of cardiac troponin I at serine 23/24 is associated with decreased interaction between cardiac troponin I and C, reduced contractile kinetics, and acceleration of the slow phase of relaxation^57^. Accordingly, we observed an increase of phospholamban phosphorylation at serine 16 residue, a target for PKA/PKG cascades^51^, suggesting that EMPA may upregulate signaling pathways involved in the modulation of contractile machinery kinetics under our experimental model and conditions. Such modulation of contractile proteins not only explains why we observed a neutral effect of EMPA in the contraction phase but also would cooperate with faster Ca^2+^ decay kinetics to improve diastolic function observed in EMPA-treated cardiomyocytes.

An additional consideration about the effects of EMPA on Ca^2+^ transients, particularly in Ca^2+^ decay kinetics, may involve the potential modulation of NHE1 by SGLT2i^25,58–60^. The inhibition of NHE1 by EMPA has been postulated to decrease [Na^+^]_i_^25,58^ and thus change chemical gradients for Na^+^:Ca^2+^ exchange between cytosol and mitochondria and between cytosol and extracellular fluid^27,28^. However, Chung and colleagues^61^ found that EMPA, over a wide range of concentrations, does not inhibit NHE1 activity in isolated rat ventricular cardiomyocytes or Langendorff-perfused beating mouse hearts^61^. In this controversial scenario, we evaluated how Ca^2+^ transients would be affected by the NHE1 inhibitor cariporide alone or in combination with EMPA. Unexpectedly, acute treatment with cariporide did not affect diastolic Ca^2+^ levels (i.e., baseline calcium levels) but enhanced Ca^2+^ transient peaks and improved Ca^2+^ decay and relaxation kinetics. Similarly, Li et al.^62^ observed that cariporide induced higher Ca^2+^ transients in isolated cardiomyocytes from healthy adult mice without affecting diastolic Ca^2+^ levels. This controversy might be explained by the simultaneous effects of cariporide on lowering [Na^+^]_i_ and pHi. As postulated by Mattiazzi and colleagues, acidosis might affect Ca^2+^ handling and, thus, contraction-relaxation dynamics via protein phosphatase-1 inhibition, leading to increased phosphorylation of different proteins, including Ca^2+^/calmodulin-dependent kinase II (CaMKII) and PLN at threonine 17 residue^63^. Regardless of the underlying mechanism, we noticed that EMPA-mediated Ca^2+^ decay and relaxation kinetics improvements persisted even in cariporide-treated cells. Albeit an inhibition of NHE1 by EMPA may not be ruled out in the present study, our results suggest that SGLT2 inhibitors might also recruit an additional NHE1-independent mechanism, which is supported by the observation of EMPA-induced increased PS16-PLN.

In summary, the present study demonstrates that EMPA influences electrical properties in rat and human cardiomyocytes, reducing arrhythmogenic events, enhancing [Ca^2+^]_i_ transient recovery, and improving relaxation kinetics, at least in part by an NHE1-independent pathway. Therefore, our study provides further evidence that the cardioprotection conferred by SGLT2 inhibitors may partially be attributed to its cardiomyocyte electrophysiological effects.

## Methods

### Animals

The study protocol was approved by the Commission of Ethics in the Use of Animals of the Medical School of the University of Sao Paulo (protocol #1150/2018). All experiments were performed in accordance with the ethical principles of animal research of the Brazilian College of Animal Experimentation, and all methods were reported in accordance with ARRIVE guidelines. Female and male Wistar rats (2 months old) weighing 200–250 g were obtained from the University of São Paulo Medical School, São Paulo, SP, Brazil. Rats at a female:male ratio of 2:1 were randomly mated in one cage. After 4 days, the female rats were individually placed in other cages until they gave birth at 22 days’ gestation. The animals were maintained in a temperature- and humidity-controlled environment with a 12-h dark/light cycle at the Heart Institute (InCor) animal facility. Food and water were supplied ad libitum.

### Rat neonatal cardiomyocytes isolation and culture

Ventricular cardiomyocytes were obtained from 1- to 2-day-old neonatal Wistar rat hearts (n = 137) as previously described^64^ with slight modifications. Briefly, the animals were euthanized by decapitation, and the hearts were excised and washed in an ice-cold ADS solution (ddH_2_O supplemented with 6.8 g/l NaCl, 4.76 g/l HEPES, 0.12 g/l NaH_2_PO_4_, 1 g/l Glucose, 0.4 g/l KCl, and 0.1 g/l MgSO_4_). The ventricles were minced, and tissue pieces were dissociated in an ADS solution containing collagenase type II (0.4 mg/ml; Worthington Biochemical Corporation, New Jersey, USA) and pancreatin (0.2 mg/ml) at 37 °C with shaking for 10 min. This digestion procedure was repeated 5–6 times. For each digestion cycle, the supernatant was removed, centrifuged at 230×*g* for 5 min, and suspended in culture medium [low-glucose DMEM and 199/EBSS (4:1) supplemented with 10% horse serum (HRS), 5% newborn calf serum (NBCS), 50 U/ml penicillin–streptomycin, and 1% sodium pyruvate; all from Thermo Fisher Scientific, Waltham, MA, USA]. After that, the cells were preplated in 100-mm culture dishes for 45 min to reduce the fibroblast content. The supernatant, after preplating, was centrifuged at 230×*g* for 5 min. Cells were resuspended in a culture medium with 1% bromodeoxyuridine (BrdU), counted, plated on laminin- or fibronectin-coated culture surfaces depending on the experiment, and incubated at 37 °C in a humidified atmosphere containing 5% CO_2_.

After 2 days, the culture medium was changed. Four days after cell plating, flow cytometric analyses were performed to evaluate the efficiency of the cardiomyocyte isolation protocol (Supplementary Material). Hypoxia conditions were chemically induced using 200 μM cobalt chloride (CoCl_2_) (Merck, Darmstadt, Germany) (added to new culture medium) for 4 h. Then, the cells were treated with vehicle (dimethyl sulfoxide; DMSO) or 1 µM EMPA (AdooQ Bioscience, Irvine, CA, USA) (added to new culture medium) for 24 h. The EMPA concentration (1 µM) was in the range of the typical plasma concentration during the 25 mg q.d. oral treatment of patients^65^. After that, experiments were conducted to analyze electrical potentials, mRNA (Supplementary Material), protein expression, Ca^2+^ transients, and contractility. For Ca^2+^ transients and contractility experiments, cells were incubated with the NHE1 inhibitor cariporide (10 µM) for 20 min in combination or not with EMPA. This concentration was chosen based on previous studies which show that 10 µM cariporide effectively inhibits NHE1-mediated Na^+^/H^+^ exchange in ventricular myocytes isolated from different species^58–60,66^. The study design is depicted in Supplementary Fig. S1.

### Cardiomyocytes derived from iPSC

All procedures were performed in accordance with relevant guidelines and regulations and approved by the National Institute of Cardiology ethics review board under number 27044614.3.0000.5272. The iPSCs used in this study were obtained from reprogramming erythroblasts derived from a healthy individual, as described previously^67^. Briefly, mononuclear cells were cultivated in an enrichment medium for erythroblasts, and after 12 days, cells were infected with CytoTune-iPS 2.0 Sendai Reprogramming Kit (Thermo Fisher Scientific). Then, the cells were cultured on a murine embryonic feeder layer in DMEM/F12, GlutaMAX supplement, with 20% KSR, 1% penicillin–streptomycin, 100 µM NEEA, 0.1 mM *β*-mercaptoethanol, 10 ng/ml of bFGF. After confirming pluripotent state^67^, the iPSCs were maintained as feeder-free culture by using 1% Geltrex™ LDEV-Free Reduced Growth Factor Basement Membrane Matrix (Thermo Fisher Scientific) in StemFlex medium (Thermo Fisher Scientific) with 1% penicillin–streptomycin.

The differentiation protocol of iPSCs into cardiomyocytes was based on the modulation of Wnt pathway signaling described by Lian and colleagues^68^. iPSCs were dissociated with TrypLE™ Express Enzyme (Thermo Fisher Scientific), and 4 × 10^5^ iPSCs were seeded in each well of a 48-well plate coated with 1% Geltrex™ and cultured for 2 days. The Wnt signaling was activated on day 0 of the protocol by treatment with 9 μM of CHIR99021 (Tocris) diluted in basal medium (RPMI 1640 supplemented with B-27 without insulin (Thermo Fisher Scientific)). On days 1 and 2, cells were maintained in basal medium. On days 3 and 4, cells were treated, respectively, with 10 μM and 5 μM of XAV939 (Tocris) for Wnt inhibition. On day 5, XAV939 was removed from the medium, and, finally, on day 7, the medium was changed for RPMI plus B-27 with insulin (Thermo Fisher Scientific). iPSC-derived cardiomyocytes were cultured until day 24 with medium changes every 3 days. Lactic acid 4 μM (Sigma-Aldrich) in RPMI 1640 with insulin without glucose/B-27 was added to the cell culture media at differentiation day 24 and maintained for 6 days with medium changes every 2 days. From day 28, iPSC-derived cardiomyocytes were dissociated with trypsin–EDTA, and 5 × 10^5^ cells were transferred in the central region of the 35-mm plate coated with matrigel. From day 30, cell cultures were subjected to normoxia or chemical hypoxia induced by 250 µM CoCl_2_ for 4 h, as previously established by our laboratory^69^. Then, the cells were treated with vehicle (DMSO) or 1 µM EMPA for 24 h. After that, cells were used for action potential recordings.

### Action potential recordings

The 35-mm laminin- or matrigel-coated plates containing 5 × 10^5^ cardiomyocytes derived from neonatal rats (n = 3 for each of 3 independent experiments) or iPSCs (n = 3 for each of 2 cardiac differentiation), respectively, were transferred to the stage of an inverted microscope (Nikon Instruments Inc., Melville, NY, USA). The action potential recording method was adapted from a previously described protocol^67^. Cells were superfused with Tyrode's solution containing 150.8 mM NaCl, 5.4 mM KCl, 1.8 mM CaCl_2_, 1.0 mM MgCl_2_, 11 mM D-glucose, and 10 mM HEPES (pH 7.4 adjusted with NaOH). The superfusion was carried out at 37.0 ± 0.5 °C using a temperature controller (Harvard Apparatus) saturated with oxygen at a perfusion flow rate of 0.5 ml/min (Minipuls 3). The transmembrane potential was recorded using glass microelectrodes (40–80 MΩ DC resistance) filled with 2.7 M KCl connected to a microelectrode amplifier (MultiClamp 700B; Molecular Devices, USA). Amplified signals were digitized (1440 Digidata A/D interface) and stored on a computer for future analysis using LabChart 7.3 software (ADInstruments). Paced (1 Hz) and/or spontaneous action potentials records were acquired. The following parameters were recorded: resting membrane potential (RMP), action potential amplitude (APA), dV/dt_max_, and APD at 30, 50, and 90% repolarization (APD30, APD50, and APD90). For the spontaneous action potentials records, beating frequency was also evaluated (Supplementary Material). Measures were obtained automatically. In rat cardiomyocytes, APDs were corrected by the beat rate. In human iPSC-derived cardiomyocytes, action potential recordings were analyzed from ventricle-like cells, classified by the following parameters: dV/dt_max_ > 10 V/s and APD30/APD90 > 0.3^70^. The starting point of the action potential was determined by the software as a 10% variation in voltage from the maximum diastolic potential.

### Cytosolic calcium transient analysis

Calcium transient recording experiments were performed as previously described^64^ with some modifications. Briefly, 3 × 10^5^ cardiomyocytes derived from neonatal rats were plated on laminin-coated glass coverslips (25 × 25 mm; Corning, New York, NY, USA) inside 60 mm culture plates (n = 3). Cells were moved to a fresh medium supplemented with 3 μM Fura-2 AM (F1201, Thermo Fisher Scientific) and incubated for 15 min at 37 °C. The medium was refreshed again with a medium free of Fura-2 AM, and the coverslip was transferred to a recording chamber. The cardiomyocytes were visualized using a microscope (Nikon Instruments Inc., Melville, NY, USA) equipped with a MyoCam-S camera (Milton, MA, USA). All the recordings were performed under electrical stimulation at 1 Hz for 4 ms and 10 V (IonOptix MyoPacer EP—Field Stimulator, Milton, MA, USA). The range in fluorescence emission wavelength was 340–380 nm, and the fluorescence signal was collected with a photomultiplier tube via the × 40 oil objective during continuous excitation at 510 nm with a 75-W Xenon lamp. The recordings were assessed by an expert blinded to the experimental groups. Data were processed and analyzed using IonWizard (Core and Analysis—IonOptix). The raw traces were filtered to reduce noise, and representative peaks from each cell were selected. Measurements of [Ca^2+^]_i_ transient amplitude, time to peak, and time to 50% relaxation (DT50) were performed. Mean curves of [Ca^2+^]_i_ transients for each experimental group were created by aligning the fluorescence signals of each cell recorded and calculated by the average of the fluorescence per timepoint.

### Contractility analysis

To measure contractile properties, cardiomyocytes derived from neonatal rats (1 × 10^4^ cells/well) were seeded onto laminin-coated 96-well plates (n = 3). Cells were incubated in a thermostatic chamber (21% O_2_ and 5% CO_2_ at 37 °C) to maintain physiologic conditions. The cardiomyocytes derived from neonatal rats were visualized using EVOS Cell Imaging System (ThermoFisher Scientific), equipped with a 20 × objective. Cells were allowed to stabilize for at least 10 min before any recordings. All the recordings were performed under electrical stimulation at 1 Hz for 20 ms and 20 V (IonOptix C-Pace EP—Cell Culture Stimulator, Milton, MA, USA) and obtained as previously described^71^. Movie images of beating cardiomyocytes were acquired with a duration of 10 s for each position, at 30 frames per second, 2048 × 1536 pixel resolution, and a pixel size of 0.307 μm/pixel with a depth of 8 bits. Data were processed and analyzed using Contractionwave, an open-source software for large-scale analysis of cardiomyocyte contraction^72^, allowing us to determine the contraction and relaxation times (CT and RT, respectively).

### SDS-PAGE and immunoblotting

Cardiomyocytes were seeded onto 35-mm laminin-coated plates at 10^5^ cells/cm^2^. The cells were washed 3 times in an ice-cold PBS buffer (150 mM sodium chloride, 2.8 mM monobasic sodium phosphate, 7.2 mM dibasic sodium phosphate, pH 7.4). Subsequently, the cells were lysed in RIPA lysis buffer (10 ×) containing protease inhibitors (1 mM phenylmethanesulfonyl fluoride, 1 µg/ml aprotinin, 1 µg/ml leupeptin, and 1 µg/ml pepstatin) and phosphatase inhibitor cocktails 2 and 3 (1:100 dilution). The cell lysate was mixed by vortexing for 30 s and centrifuged at 15,800×*g* for 15 min at 4 °C. The supernatant was isolated and stored at − 80 °C. Protein concentration was determined using the Pierce BCA protein assay kit (Thermo Fisher Scientific) following the manufacturer's instructions. Then, samples of the cardiomyocyte proteins were solubilized in the Laemmli sample buffer and separated by SDS-PAGE using 7.5% polyacrylamide gels. For immunoblotting, proteins were transferred to PVDF membranes (Millipore Immobilon-P, Millipore, Bedford, MA) and incubated with a blocking solution containing 5% skim milk or BSA and 0.1% Tween 20 in PBS (pH 7.4) for 1 h to block nonspecific antibody binding, followed by overnight (4 °C) incubation with specific monoclonal antibodies against HIF-1α (D2U3T) (Cell Signaling Technology, 14,179, 1:1000 dilution), phospholamban (PLN) [2D12] (Abcam, ab2865, 1:1,000 dilution), actin (Abcam, ab179467, 1:5,000 dilution) or a polyclonal antibody against PLN (phospho S16) (Abcam, ab15000, 1:1,000 dilution). The PVDF membranes were washed five times in a blocking solution and incubated for 1 h at room temperature with the horseradish peroxidase-conjugated immunoglobulin secondary antibody (Jackson ImmunoResearch Laboratories, Inc, 1:2,000 dilution). After washing five times with blocking solution and twice in PBS (pH 7.4), the PVDF membranes were incubated for 1 min with an enhanced chemiluminescence detection (ECL) system (Cytiva, Marlborough, MA, USA) for visualization of the bound antibodies. The visualized bands were digitized using ImageScanner III (Cytiva) and quantified using ImageJ software (National Institutes of Health, Bethesda, MD). The unedited gel images are included within the supplementary material of the manuscript.

### Statistical analyses

The results are reported as the mean ± standard error of the mean (SEM). Data normality was tested (Shapiro–Wilk test or Kolmogorov–Smirnov test). For the analysis of action potential, statistical significance was determined using unpaired *t*-test or Mann–Whitney test. For analyses of proportions, Fisher's exact test was used. For calcium transients, contractility, and immunoblot analyses, statistical significance was determined using one-way ANOVA followed by a post hoc Tukey analysis or Kruskal–Wallis test, both with multiple comparisons with 95% confidence. A *P* value < 0.05 was used to indicate significance. All statistical analyses were performed using GraphPad Prism version 8.0 (GraphPad Software, La Jolla, CA).

## Supplementary Information

Supplementary Information.

## Data Availability

All data generated or analyzed during this study are included in this published article and its Supplementary Information files.
