# Supplementary material for: Empagliflozin reduces arrhythmogenic effects in rat neonatal and human iPSC-derived cardiomyocytes and improves cytosolic calcium handling at least partially independent of NHE1
Source: Sci Rep. 2023 May 29;13:8689. doi: 10.1038/s41598-023-35944-5 (PMC10226982; doi:10.1038/s41598-023-35944-5)
Supplement: Supplementary file 1 — Supplementary Information. [file 41598_2023_35944_MOESM1_ESM.pdf]

SUPPLEMENTARY MATERIAL

Empagliflozin reduces arrhythmogenic effects in rat neonatal and human iPSC-derived cardiomyocytes and improves cytosolic calcium handling at least partially independent of NHE1

Danúbia Silva dos Santos[1], Lauro Thiago Turaça[1], Keyla Cristiny da Silva Coutinho[2], Raiana Andrade Quintanilha Barbosa[2,3], Juliano Zequini Polidoro[1], Tais Hanae Kasai Brunswick[2,4], Antonio Carlos Campos de Carvalho[2,4], Adriana Castello Costa Girardi[1]

Affiliations

[1]Laboratório de Genética e Cardiologia Molecular, Instituto do Coração (InCor), Hospital das Clínicas HCFMUSP, Faculdade de Medicina, Universidade de São Paulo, São Paulo, Brasil

[2]Carlos Chagas Filho Institute of Biophysics, Federal University of Rio de Janeiro, Rio de Janeiro, RJ, Brazil

[3]Center of Cellular Technology, National Institute of Cardiology, Rio de Janeiro, Brazil

[4]National Center of Structural Biology and Bioimaging (CENABIO), Federal University of Rio de Janeiro, Rio de Janeiro, RJ, Brazil

Flow cytometric analyses

For the studies, cardiomyocytes were seeded onto 35-mm laminin-coated plates at 10^5 cells/cm^2. On the 4th day (before being subjected to normoxia or chemical hypoxia), cells were dissociated with 0.25% trypsin-EDTA (Thermo Fisher Scientific). For extracellular staining, the cells were blocked with 0.5% bovine serum albumin (BSA) in PBS and 1 µl/ml Fc Block (550270; BD Biosciences) for 20 min at 4°C. Subsequently, the cells were stained with antibodies specific for CD31 (BD Biosciences, 555025, 1:100 dilution) and CD90 (BD Biosciences, 561409, 1:100 dilution) for 30 min at 4°C. For intracellular staining, cells were fixed in 4% paraformaldehyde for 20 min at room temperature and permeabilized with 0.3% Triton X-100 in PBS for 30 min. The cells were then blocked with 0.5% BSA in PBS and stained with Alexa Fluor 647 anti-cardiac troponin T (BD Biosciences, 565744, 1:200 dilution) for 30 min at room temperature. Then, the cells were selected by 7-amino-actinomycin D (7AAD) (Thermo Fisher Scientific) staining. All data were acquired with a BD Accuri C6 and analyzed using FlowJo software version 10.

Analysis of the efficiency of the cardiomyocyte isolation protocol and HIF-1α expression.

After 24 h in culture, rat cardiomyocytes adhered to the coated dishes (Supplementary Figure S2A) and started spontaneous contractions. On day 4, we observed a near-confluent cell monolayer (Supplementary Figure S2B). Before beginning the experiments, we evaluated the efficiency of the cardiomyocyte isolation protocol by determining the expression of CD31, CD90, and cardiac troponin T proteins commonly used to identify endothelial cells (1), cardiac fibroblasts (2), and cardiomyocytes (3), respectively. As seen in Supplementary Figure S2C-E, approximately 90% of cells were positive for cardiac troponin T, whereas low expression of CD31 and CD90 was detected. These results confirm the efficient cardiomyocyte isolation protocol.

On day 4, rat cardiomyocytes were treated with $CoCl_2$ to induce hypoxia. $CoCl_2$ is reported to mimic hypoxia due to stabilizing the expression of hypoxia-inducible factor-1α (HIF-1α) (4). Consistent with this study, we found that HIF1-α protein abundance was enhanced in rat cardiomyocytes treated with $CoCl_2$ compared with untreated cells (Supplementary Figure S3).

Figure S1. Experimental design.

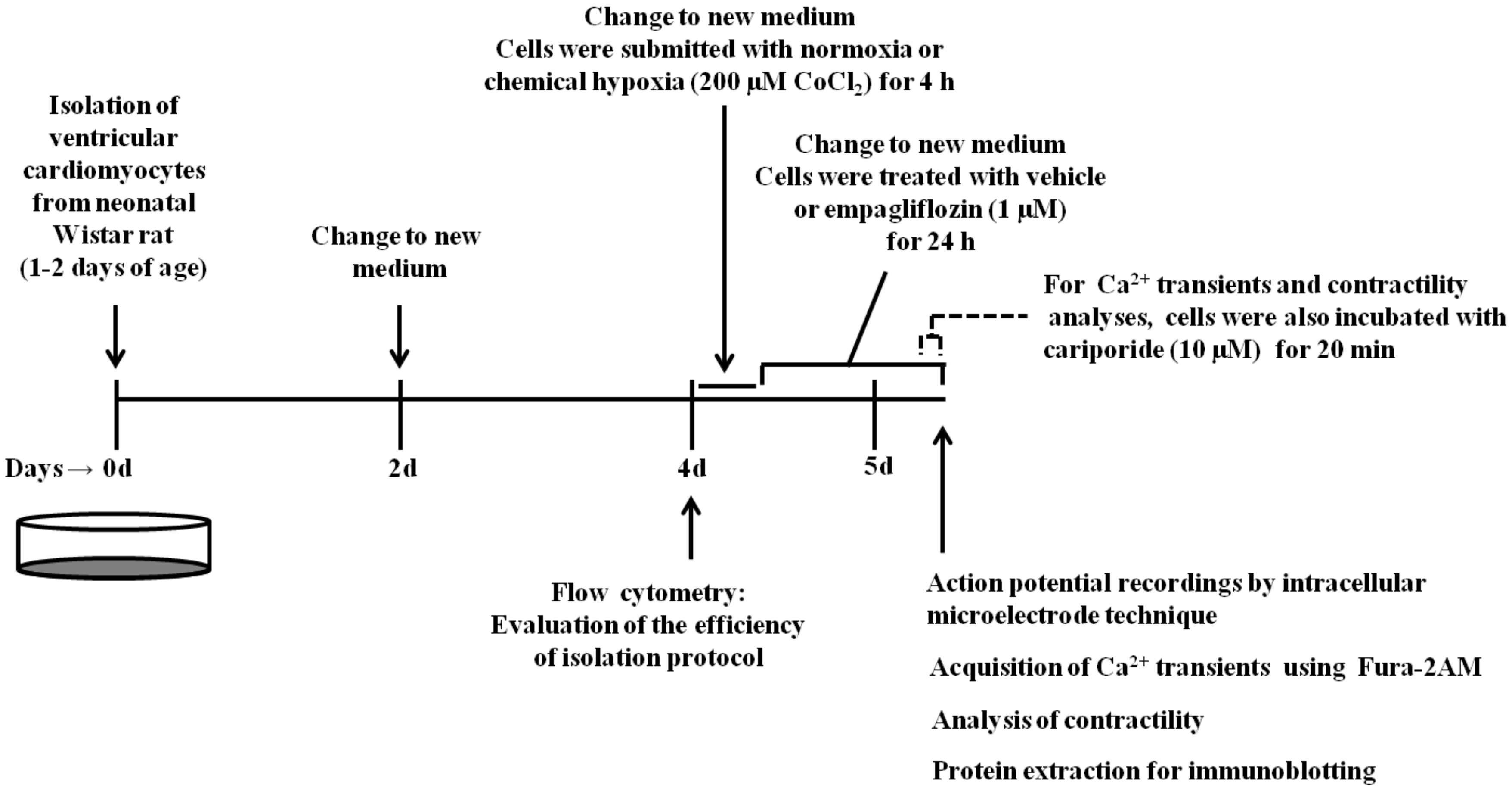

Figure S2.

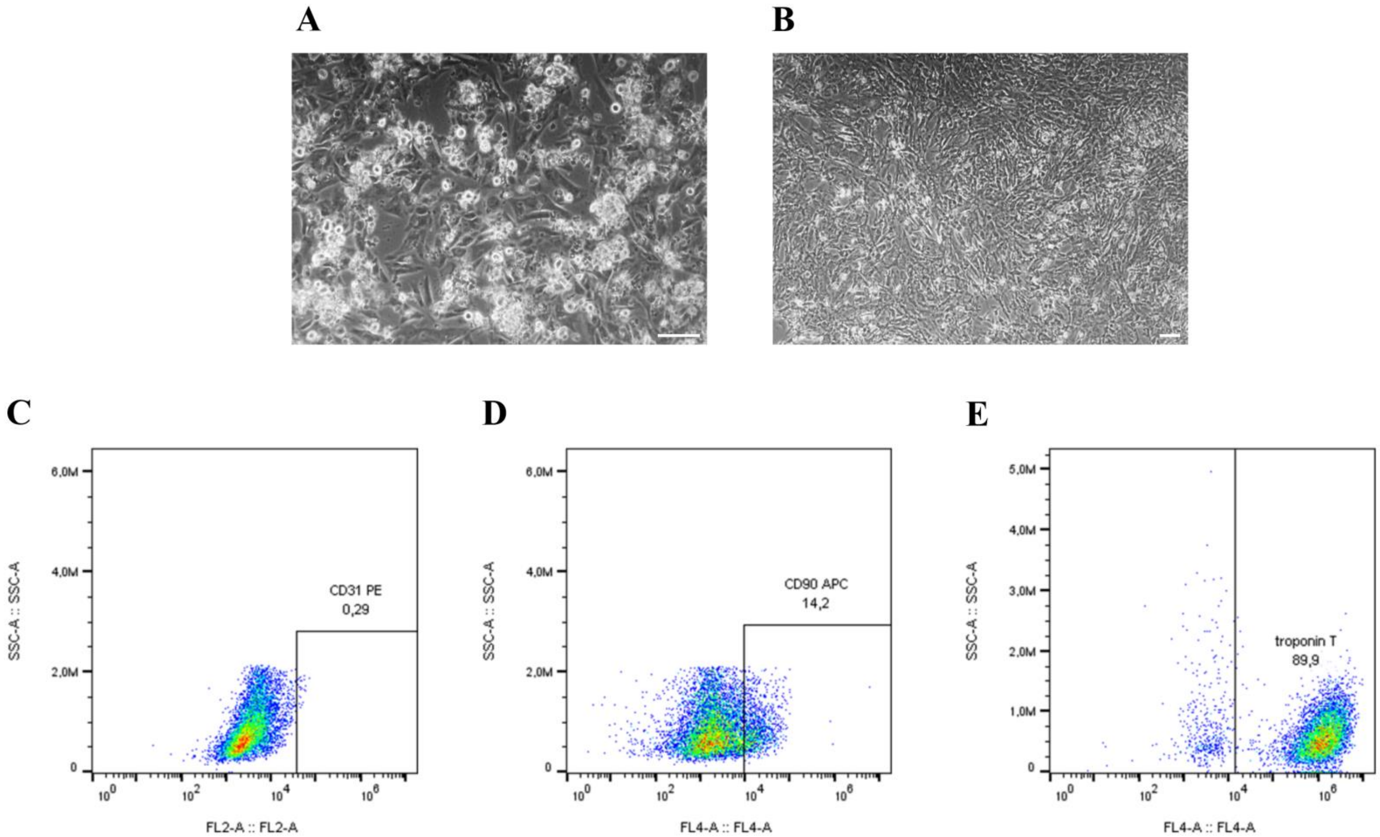

<u>Figure S2.</u> Analysis of the efficiency of the cardiomyocyte isolation protocol. (A-B) Representative images of the ventricular cardiomyocytes derived from neonatal rats in culture: **(A)** after 24 h, cardiomyocytes adhered to the coated dishes; **(B)** after 4 days, formation of a near-confluent cells monolayer. Scale bar: 100 µm. **(C-E)** The culture was evaluated by expression of **(C)** CD31 PE (endothelial marker), **(D)** CD90 APC (cardiac fibroblasts marker), and **(E)** troponin T Alexa Fluor 647 (cardiomyocytes marker). The representative dot blots show: on the x-axes, the percentage of positive cells for each cell marker; on the y-axes, the lateral dispersion (SSC-A).

Figure S3.

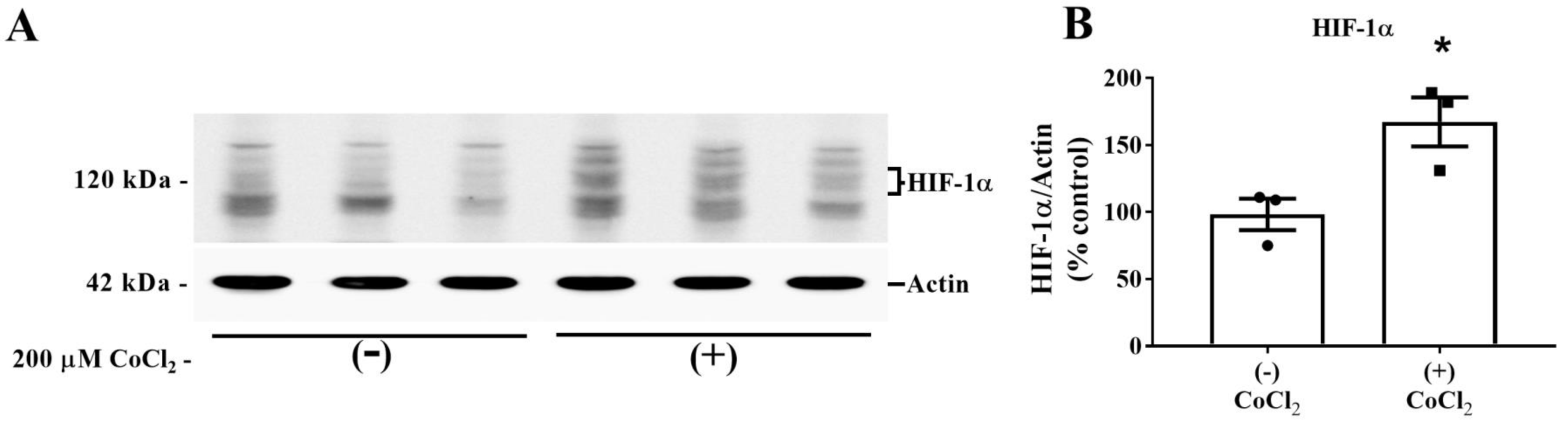

<u>Figure S3.</u> Analysis of the expression of hypoxia-inducible factor-1α (HIF-1α). (A) Representative immunoblot of HIF-1α expression in isolated rat neonatal cardiomyocytes treated (+) or not (-) with 200 μM cobalt chloride to induce hypoxia for 4 hours. Actin was used as an internal control. **(B)** Graphical representation of the relative expression of HIF-1α in cardiomyocytes treated with 200 μM cobalt chloride (+) or not (-). The points represent individual measurements, and the bars represent the mean ± SEM ($n = 3$). The significance of differences was determined using the unpaired *t*-test. *$P < 0.05$ vs. control.

Figure S4.

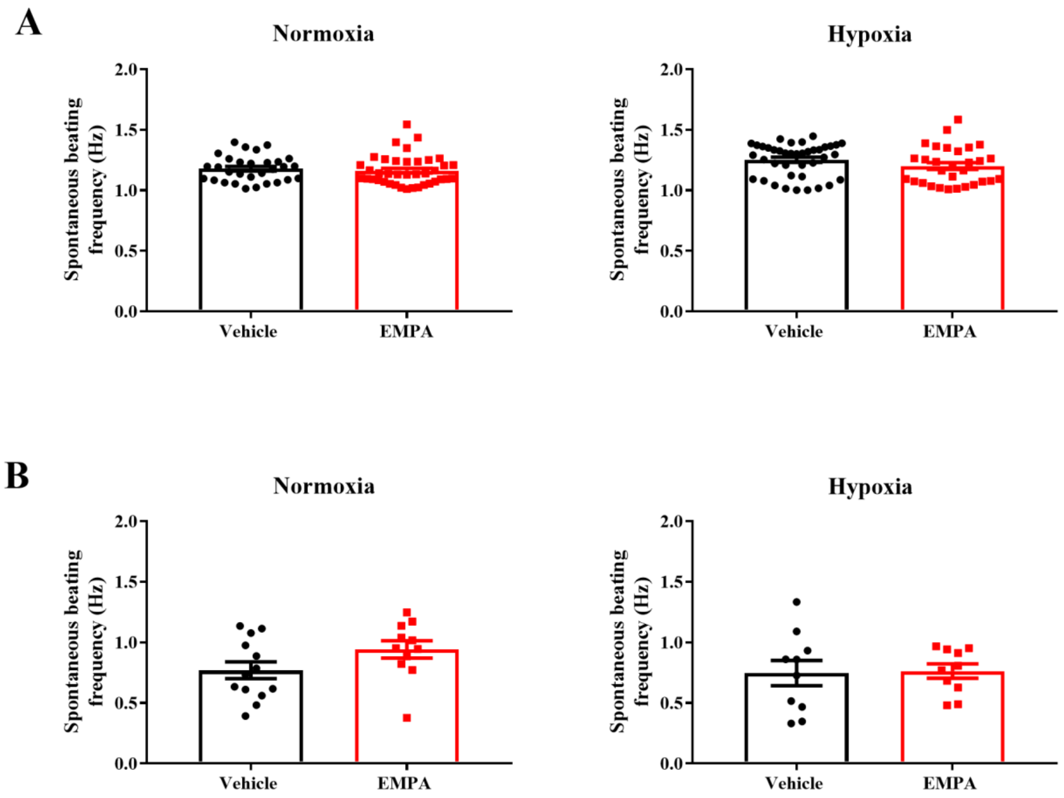

<u>**Figure S4.**</u> **Analysis of the spontaneous beating frequency of action potential.** Spontaneous beating frequency of action potential acquired from rat neonatal cardiomyocytes **(A)** or iPSCs-derived cardiomyocytes **(B)** subjected to normoxia or chemical hypoxia and treated with vehicle or EMPA. Statistical analysis was performed using the unpaired *t*-test or Mann-Whitney test. The points represent individual measurements, and the bars are the mean ± SEM (rat neonatal cardiomyocytes, *n* = 30-41; iPSCs-derived cardiomyocytes, *n* = 10-13).

Figure S5. Full unedited gel for Figure 8 (PS16-PLN)

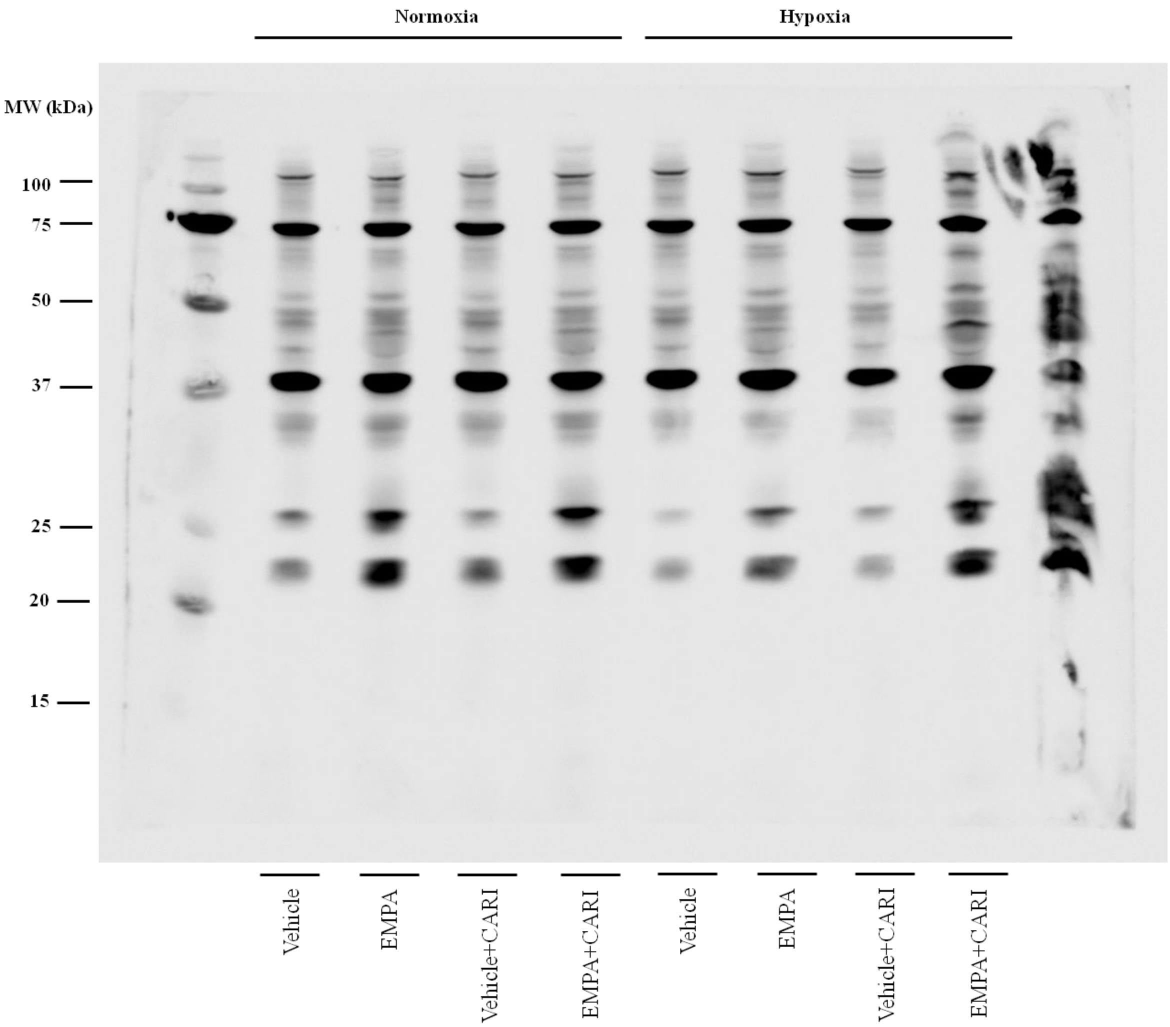

Figure S5. Full unedited gel for Figure 8 (total PLN)

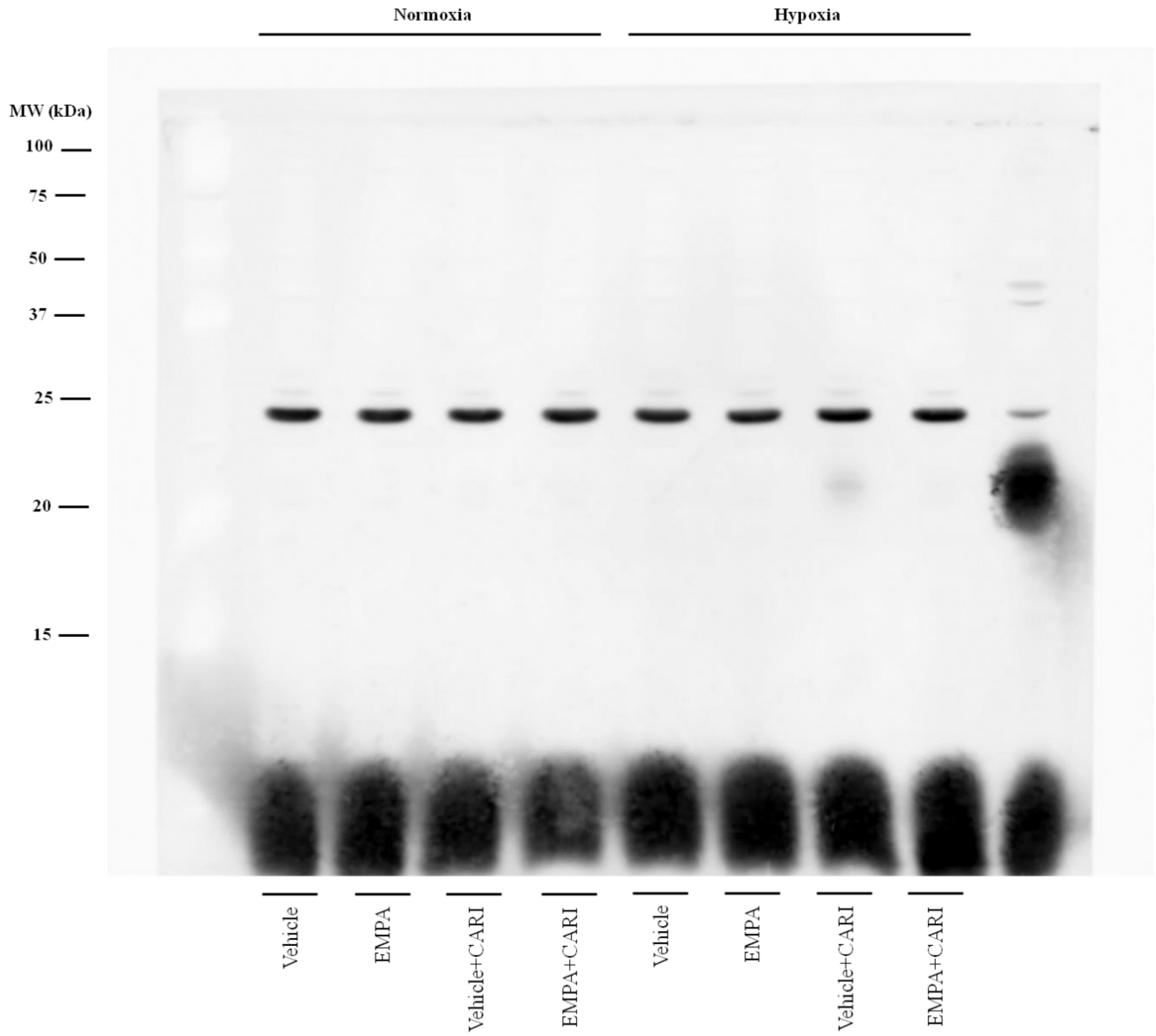

Full unedited gel for Figure 8 (β-actin)

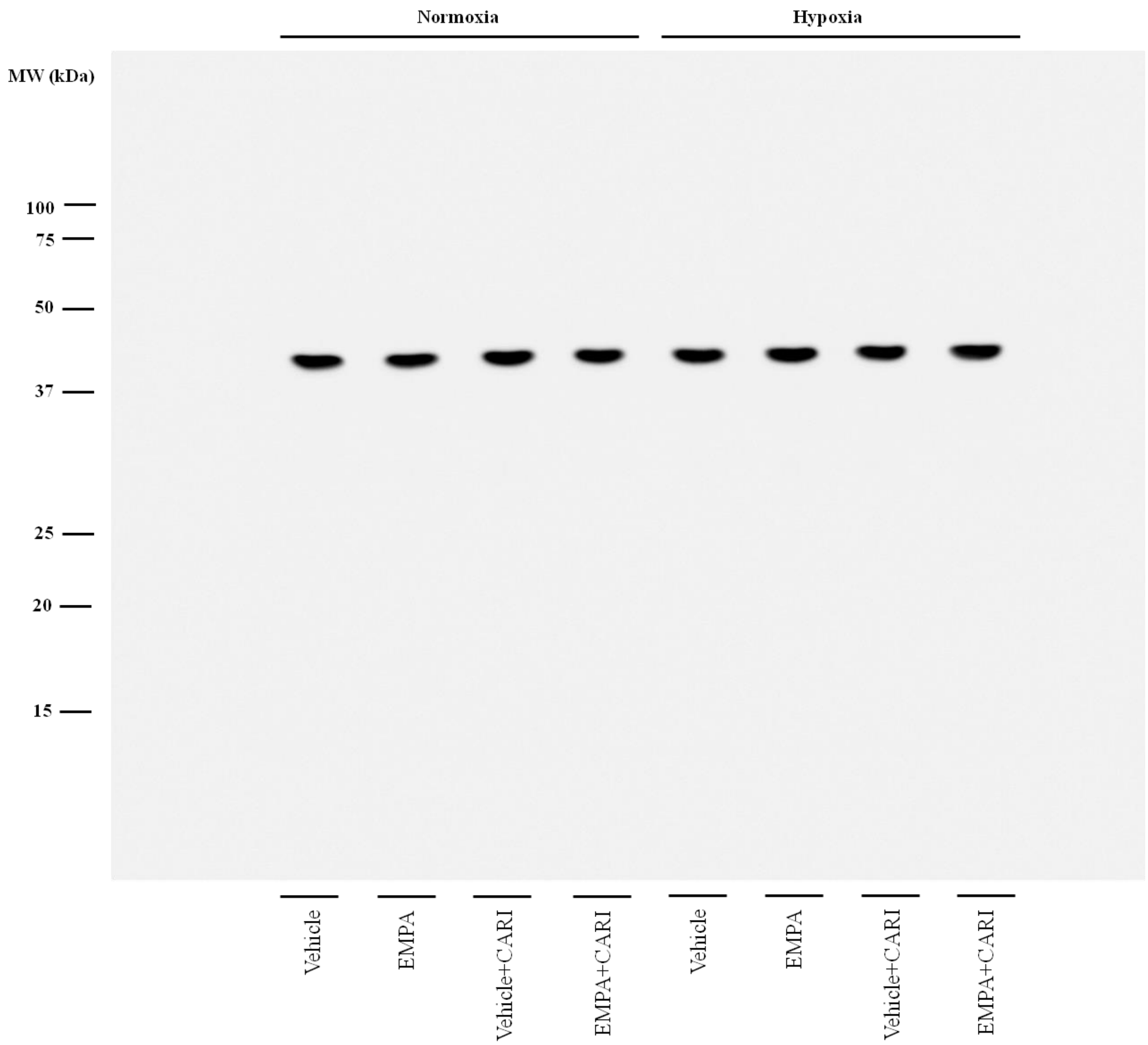

Figure S6. Full unedited gel for Supplemental Figure S3 (HIF-1α)

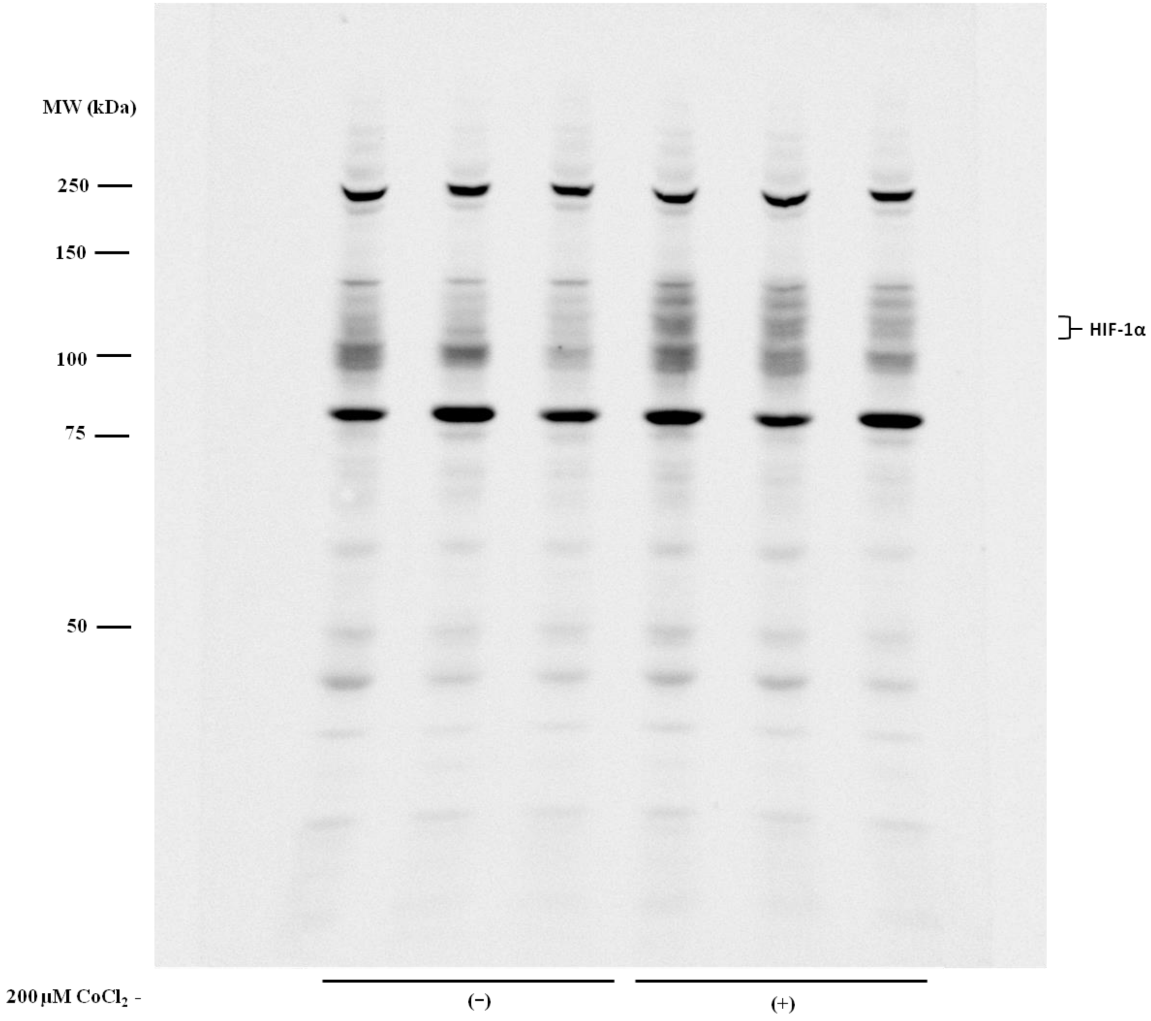

Figure S6. Full unedited gel for Supplemental Figure S3 (Actin)

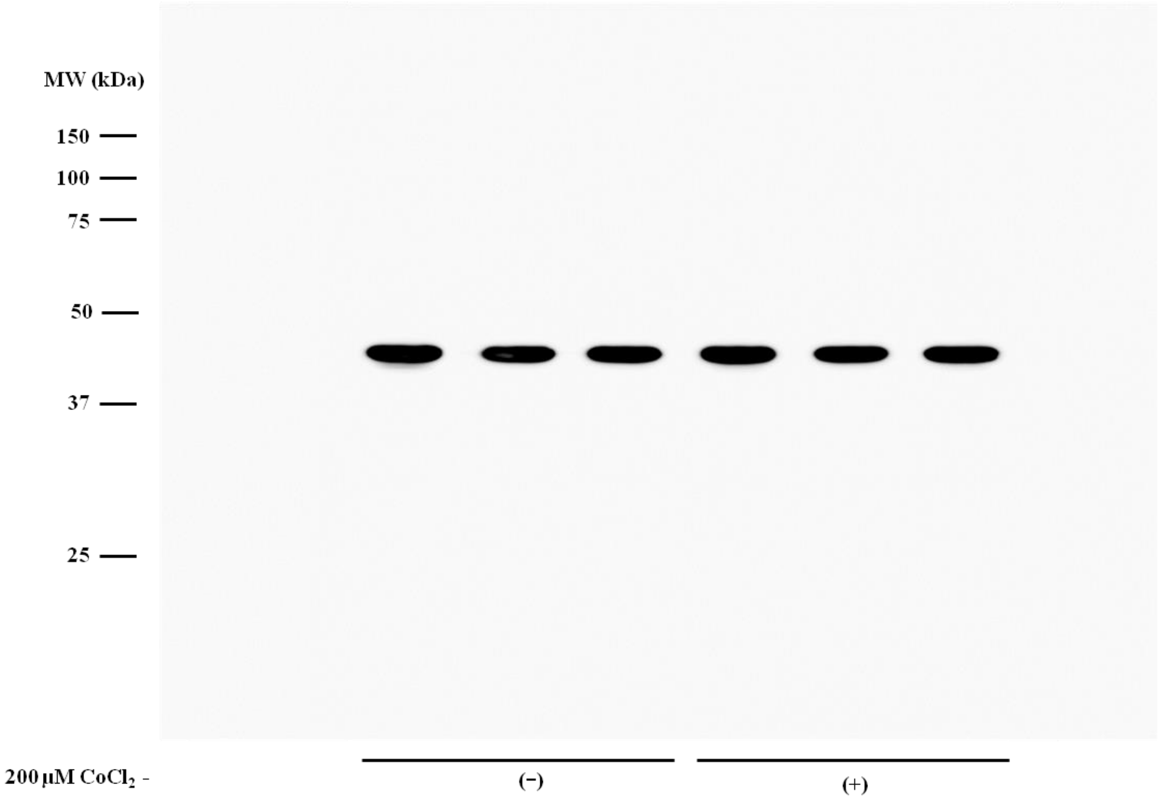

References

1. Lertkiatmongkol P, Liao D, Mei H, Hu Y, Newman PJ. Endothelial functions of platelet/endothelial cell adhesion molecule-1 (CD31). Curr Opin Hematol. 2016;23(3):253-9.
2. Moore-Morris T, Guimarães-Camboa N, Yutzey KE, Pucéat M, Evans SM. Cardiac fibroblasts: from development to heart failure. J Mol Med (Berl). 2015;93(8):823-30.
3. Forough R, Scarcello C, Perkins M. Cardiac biomarkers: a focus on cardiac regeneration. J Tehran Heart Cent. 2011;6(4):179-86.
4. Muñoz-Sánchez J, Chánez-Cárdenas ME. The use of cobalt chloride as a chemical hypoxia model. J Appl Toxicol. 2019;39(4):556-70.
